# Live imaging of adult zebrafish cardiomyocyte proliferation *ex vivo*

**DOI:** 10.1242/dev.199740

**Published:** 2021-09-13

**Authors:** Hessel Honkoop, Phong D. Nguyen, Veronique E. M. van der Velden, Katharina F. Sonnen, Jeroen Bakkers

**Affiliations:** 1 Hubrecht Institute-KNAW and Utrecht University Medical Center, Utrecht 3584CT, The Netherlands; 2 Department of Pediatric Cardiology, Division of Pediatrics, University Medical Center Utrecht, Utrecht 3584EA, The Netherlands

**Keywords:** Cardiac, Live-imaging, Proliferation, Regeneration, Sarcomere, Zebrafish

## Abstract

Zebrafish are excellent at regenerating their heart by reinitiating proliferation in pre-existing cardiomyocytes. Studying how zebrafish achieve this holds great potential in developing new strategies to boost mammalian heart regeneration. Nevertheless, the lack of appropriate live-imaging tools for the adult zebrafish heart has limited detailed studies into the dynamics underlying cardiomyocyte proliferation. Here, we address this by developing a system in which cardiac slices of the injured zebrafish heart are cultured *ex vivo* for several days while retaining key regenerative characteristics, including cardiomyocyte proliferation*.* In addition, we show that the cardiac slice culture system is compatible with live timelapse imaging and allows manipulation of regenerating cardiomyocytes with drugs that normally would have toxic effects that prevent their use. Finally, we use the cardiac slices to demonstrate that adult cardiomyocytes with fully assembled sarcomeres can partially disassemble their sarcomeres in a calpain- and proteasome-dependent manner to progress through nuclear division and cytokinesis. In conclusion, we have developed a cardiac slice culture system, which allows imaging of native cardiomyocyte dynamics in real time to discover cellular mechanisms during heart regeneration.

## Abstract (precis)

**Summary:** A method that allows live imaging of proliferating cardiomyocytes during zebrafish heart regeneration in their native tissue context.

## INTRODUCTION

Cardiomyocyte turnover in the adult mammalian heart is typically very low (Bergmann et al., 2009; Senyo et al., 2013), which hampers cardiac regeneration after injury. The limited proliferative capacity of mammalian cardiomyocytes can be stimulated by activation of ErbB2 signaling, inactivation of Hippo signaling or by inducing metabolic reprogramming of cardiomyocytes, all of which result in an improved cardiac function after injury (Heallen et al., 2013; Puente et al., 2014; D'Uva et al., 2015; Cardoso et al., 2020; Magadum et al., 2020).

Zebrafish hold a remarkable capacity to regenerate their hearts after injury (Poss et al., 2002). This is achieved by a process in which surviving cardiomyocytes located in a region close to the injury, also called the border zone, proliferate to restore the damaged myocardium (Jopling et al., 2010; Kikuchi et al., 2010). Cardiomyocyte proliferation in the adult zebrafish heart can be induced by activation of Nrg1, vitamin D or Klf1 signaling, and involves changes in energy metabolism (Gemberling et al., 2015; Han et al., 2019; Honkoop et al., 2019; Fukuda et al., 2020; Ogawa et al., 2021); however, very little is known about cellular processes and mechanisms within proliferating adult cardiomyocytes.

Historically zebrafish embryos have been an excellent tool for live *in vivo* imaging due to the transparency of the embryo and the easy generation of transgenic tools to mark and manipulate cells. As a result, cardiomyocyte behavior has been studied extensively in the developing zebrafish embryo (Auman et al., 2007; Rohr et al., 2008; Smith et al., 2008; Lou et al., 2011; Uribe et al., 2018; Priya et al., 2020). Nevertheless, detailed studies of cardiomyocytes in the adult heart have been hampered by the lack of appropriate live-imaging tools due to the opacity of adult organs. Hence, current knowledge on zebrafish heart regeneration is based on snapshots taken at key timepoints after injury, thereby losing temporal information that is paramount for an in-depth understanding of the cellular events underlying proliferation.

In order to resolve this problem, primary adult cardiomyocytes (Sander et al., 2013) and cardiospheres (Zeng et al., 2017) from the zebrafish heart have been cultured successfully. Yet these systems lack the context of an injury, maturity of cardiomyocytes and the complexity of other cell types present in the heart. Whereas the embryonic zebrafish heart can be successfully explanted and cultured for further studies *ex vivo* (Noël et al., 2013), internal infarctions were observed after culturing adult hearts (Kikuchi et al., 2011). This limitation combined with a need for deep-tissue imaging has rendered this culture method insufficient for the imaging of proliferating cardiomyocytes in the adult heart. Recent advances in adult heart culture have led to new insights for cell types such as the epicardium (Cao and Poss, 2016) and coronary vessels (Yip et al., 2020) during heart regeneration. Nevertheless, efforts to image proliferating cardiomyocytes in the adult zebrafish heart have thus far not been described.

Here, we present a method for culturing of cardiac slices from the adult zebrafish heart. Cardiac slices from cryoinjured hearts retain regenerative characteristics, including cardiomyocyte proliferation. Importantly, this *ex vivo* system is compatible with live imaging. Live imaging on these cardiac slice cultures allowed us to gain novel insights into the cellular dynamics that accompany cardiomyocyte proliferation within its native tissue context. We, for example, find proteasome-mediated sarcomere breakdown during cardiomyocyte proliferation in *ex vivo* cultures of regenerating hearts.

## RESULTS AND DISCUSSION

### Cardiac slices retain characteristics of the regenerating heart

Slices of mammalian hearts have been invaluable tools for pre-clinical drug screening, as they retain electrophysiological properties of the intact heart (Kang et al., 2016; Watson et al., 2017; Perbellini et al., 2018). Based on this premise, we aimed to establish a cardiac slice culture system of the injured adult zebrafish heart to study the molecular processes of heart regeneration.

**Fig. 1.**
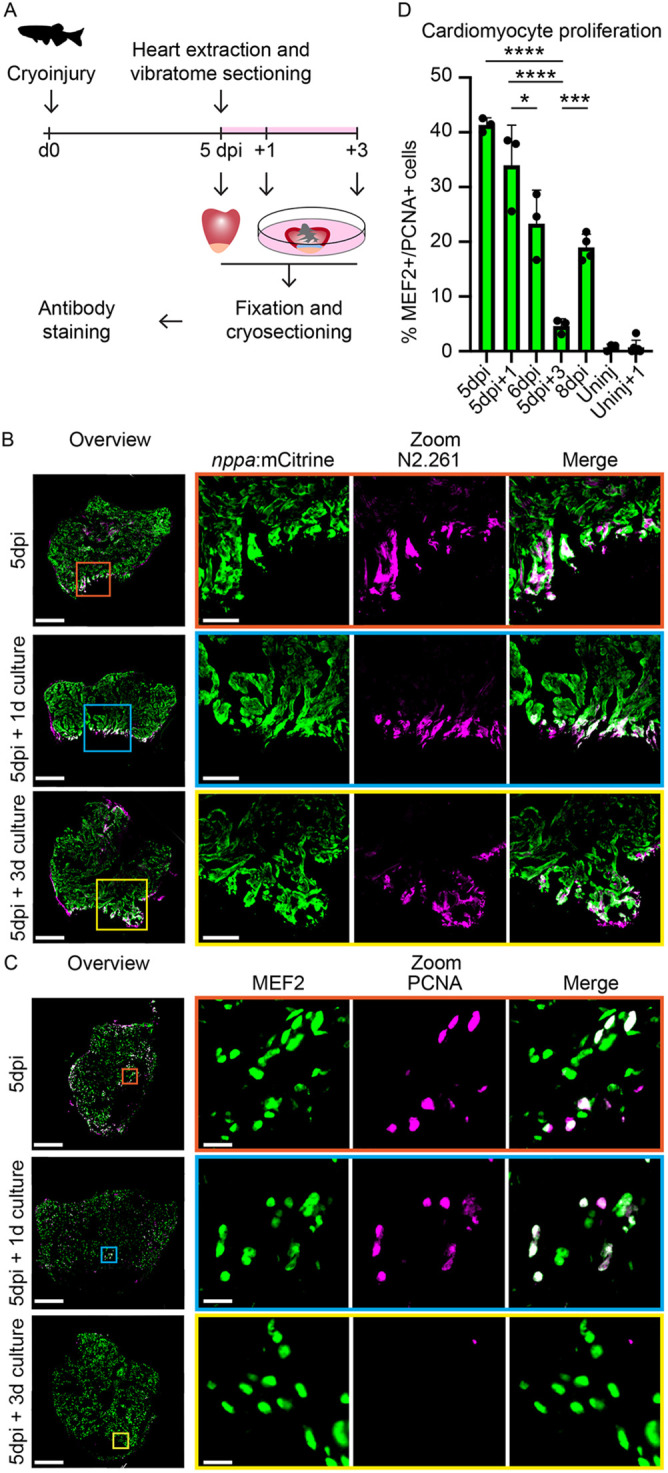
**Cardiac slices retain characteristics of the regenerating heart.** (A) Schematic representation of workflow. (B) Immunolabeling on 5 dpi whole hearts, 5 dpi cardiac slices after 1 day culture and 5 dpi cardiac slices after 3 day culture from a Tg(*nppa:mCitrine*) transgenic line. Trabecular cardiomyocytes are marked with mCitrine (green) and embryonic myosin is marked with N2.261 (magenta). Scale bars: 200 µm (overview); 50 µm (zoom). (C) Immunolabeling on 5 dpi whole hearts, 5 dpi cardiac slices after 1 day culture and 5 dpi cardiac slices after 3 day culture from a Tg(*nppa:mCitrine*) transgenic line. Cardiomyocyte nuclei are marked with MEF2 (green) and proliferating nuclei are marked with PCNA (magenta). Scale bars: 200 µm (overview) or 10 µm (zoom). (D) Quantification of MEF2^+^/PCNA^+^ cells in the border zone of injured hearts/cardiac slices and remote zone of uninjured hearts/cardiac slices. Each dot represents a result from one heart or cardiac slice. Data are mean±s.d. *P<0.05, ***P<0.001, ****P<0.0001.

To this end, we cryoinjured zebrafish hearts and extracted these at 5 days post-injury (dpi), when border zone cardiomyocytes can be identified by the expression of embryonic myosin and *nppa* and cardiomyocyte proliferation is apparent (Lepilina et al., 2006; Sallin et al., 2015). After heart extraction, the heartbeat was stopped by 2,3-butanedione monoxime (BDM) and hearts were mounted in agarose. Embedded hearts were subsequently vibratome sectioned and cardiac slices were cultured in culture medium for several days during which they maintained their morphology (Fig. 1A) (see Materials and Methods for details). TUNEL-staining revealed that, while vibratome sectioning and culturing of cardiac slices leads to some increase in cell death, this is significantly less compared with the observed widespread cell death in cultured whole hearts. Importantly, culturing cardiac slices did not result in a significant loss of cardiomyocytes (Fig. S1).

To assess the efficacy of cardiac slice culture in retaining characteristics of the injured zebrafish heart we analyzed expression of *nppa* and embryonic myosin, both of which have typical and well-characterized expression in the injured heart (Fig. 1B) (Lepilina et al., 2006; Sallin et al., 2015). We used *Tg(nppaBAC:mCitrine)* to detect *nppa* expression for which we previously showed that *nppa* expression in the ventricular trabeculated myocardium is elevated in border zone cardiomyocytes (Honkoop et al., 2019). We observed that ventricular cardiomyocytes maintained low levels of *nppa*:mCitrine after 1 and 3 days of culture, and that border zone cardiomyocytes retained elevated levels of *nppa*:mCitrine and embryonic myosin N2.261 (Fig. 1B).

Next, we investigated whether border zone cardiomyocytes also retain their proliferative capacity in the cardiac slice culture system. We therefore co-stained cultured cardiac slices and freshly extracted hearts with MEF2, which marks cardiomyocyte nuclei, and PCNA, which marks DNA replication, and quantified the percentage of PCNA^+^ cardiomyocytes. Importantly, the percentage of PCNA^+^ cardiomyocytes in freshly extracted hearts or in cardiac slices after 24 h of culturing was not significantly different (Fig. 1C,D), indicating that cardiomyocyte proliferation is maintained in the cardiac slice culture system. As we did not observe a significant increase in PCNA^+^ cardiomyocytes in cardiac slices of uninjured hearts upon culturing, we conclude that the cardiomyocyte proliferation we observed is a response to the cryoinjury and not a response to the sectioning of the hearts or culture conditions (Fig. 1D). After 3 days of culture, the percentage of PCNA^+^ cardiomyocytes in cardiac slices was strongly reduced, which may be due to either inhibitory components in the culture medium or the lack of stimuli to maintain cardiomyocyte proliferation. EdU incorporation during cardiac slice culture confirmed that cardiomyocytes were actively cycling during the first 24 h of culture, after which proliferation in the slices ceased. Cell proliferation was also observed in non-cardiomyocytes (Fig. S2). Future studies should focus on optimizing culture conditions by modifying the media composition or the matrix in which the slices are embedded to prolong the time that cardiomyocyte proliferation is maintained.

Together, these results demonstrate that zebrafish cardiac slice cultures retain characteristics of the injured heart, including cellular identity of border zone cardiomyocytes and the persistence of proliferation in the zebrafish border zone. Hence, zebrafish cardiac slice cultures provide unique opportunities in which adult cardiomyocyte proliferation can be studied within its native tissue context.

### Live imaging of cardiomyocyte proliferation in cardiac slices

**Fig. 2.**
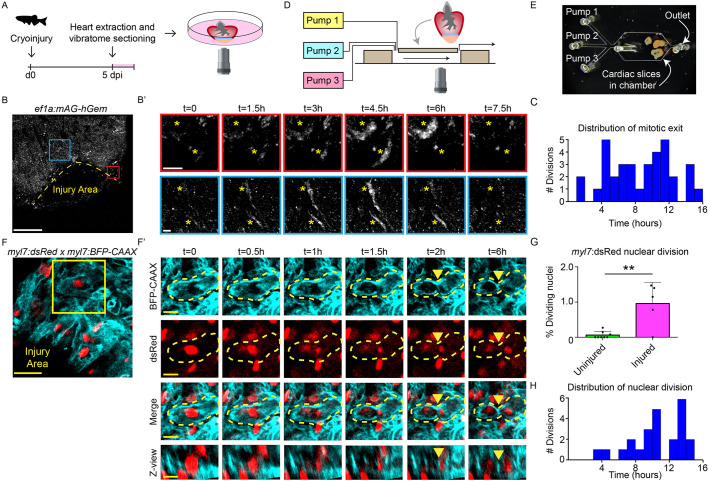
**Cardiomyocyte proliferation during live imaging in cardiac slices.** (A) Schematic representation of workflow. (B,B′) Time-lapse imaging on cardiac slices from 5 dpi *Tg(ef1α:mAg-hGem)* hearts, to visualize cells that enter G2/M phase. Yellow dotted line indicates the injury area. Asterisks mark cells in zoom-ins with dynamic geminin expression. Scale bars: 200 µm (overview); 20 µm (zoom). (C) Histogram showing distribution of mitotic exit in *Tg(EF1α:mAg-hGem)* cardiac slices over time. (D) Schematic representation of microfluidic chip design. (E) Bright-field image of cardiac slices inside the microfluidic chamber. (F,F′) Time-lapse imaging on 5 dpi cardiac slices from *Tg(myl7:dsRed;myl7:BFP-CAAX)* fish. Cardiomyocytes expressing nuclear dsRed are shown in red and membrane-bound BFP-CAAX is shown in cyan. Yellow dotted line indicates cellular outline. Yellow arrowheads indicate the membrane splitting two daughter cells. Scale bars: 25 µm (overview); 10µm (zoom). (G) Quantification of nuclear divisions during time-lapse imaging on *Tg(myl7:dsRed)* injured and uninjured slices. Each dot represents a result from one cardiac slice. Data are mean±s.d. (H) Histogram showing distribution of cardiomyocyte nuclear division. **P<0.01.

As we observed proliferation in adult zebrafish cardiac slices during culture, we addressed whether proliferation could be observed by live imaging of cardiac slices (Fig. 2A). To study this, we performed live imaging during the first 16 h of culture on a *Tg(efα:mAG-hGem)* line, which marks all cells that are in the G2/M phase of the cell cycle (Sugiyama et al., 2009). Within a cardiac slice, we observed border zone cells in which geminin levels were undetectable at the start but levels increased over time (Fig. 2B,B′, Movies 1, 2 and 3), indicating a transition from G1 to G2/M phase. This was followed by a rapid decrease in geminin signal intensity, corresponding to the completion of mitosis and re-entry into G1 phase. Furthermore, dynamic geminin expression was observed throughout the duration of the time-lapse imaging (Fig. 2C), indicating that it was not perturbed by the imaging itself. Importantly, these movies not only confirm the presence of actively cycling cells in zebrafish cardiac slices, but also show that cardiac slice cultures are compatible with live imaging.

*Ex vivo* slice cultures open up the opportunity to study cellular processes in regenerating zebrafish hearts by pharmacological perturbations that would otherwise have detrimental effect on the health of the fish because they interfere with general processes that are important in other cells and organs (e.g. energy metabolism or transcription/translation). To combine the temporal control of external perturbations with simultaneous real-time imaging (Sonnen et al., 2018), we tested whether cardiac slices can be cultured in a microfluidic chip and maintain proliferation. To address this, *Tg(EF1α:mAG-hGem)* cardiac slices were loaded onto a microfluidic chip and imaged for 16 h (Fig. 2D,E). During imaging, culture medium was flushed over the chip at a continuous flow rate. Cardiac slices within the chip survived and maintained proliferative capacity, as indicated by entry and exit of G2/M phase in cardiac slices (Fig. S3). Thus, *ex vivo* heart slice cultures in combination with microfluidics allow the analysis of the consecutive steps of zebrafish heart regeneration.

As cardiomyocyte proliferation is a key process during heart regeneration, studying this process in a temporal manner is of particular interest. To visualize mitosis and cytokinesis, we cryoinjured fish carrying both a *Tg(myl7:*DsRed*)*, which expresses a nuclear dsRed protein in all cardiomyocytes, and a *Tg(myl7:BFP-CAAX)*, which expresses a membrane-bound BFP in all cardiomyocytes, followed by time-lapse imaging of cardiac slices starting at 5 dpi. Strikingly, we were able to capture cardiomyocyte nuclear divisions in single cardiac slices followed by cytokinesis (Fig. 2F,F′,G,H). Closer inspection of individual cardiomyocytes revealed initial breakdown of the nuclear envelope at the start of mitosis. (Fig. 2F′, t=0-0.5 h, Movie 4). This is quickly followed by separation of the two new daughter nuclei (t=1-1.5 h). After nuclear division, the membrane splits the two daughter nuclei into two cells, indicating that nuclear division is followed by cytokinesis (t=1.5-6 h). This is consistent with the earlier observation that cardiomyocytes in the regenerating zebrafish heart are mainly diploid and mononucleated (González-Rosa et al., 2018). The process of nuclear envelope breakdown to the onset of cytokinesis takes ∼90 min. Importantly, nuclear divisions are induced by the cryoinjury as nuclear divisions during live imaging were rarely observed in cardiac slices from uninjured hearts (Fig. 2G). Together, these results demonstrate that cellular processes such as injury-induced proliferation can be studied live in cardiac slice cultures.

### Dynamics of sarcomere structures during cardiomyocyte proliferation

**Fig. 3.**
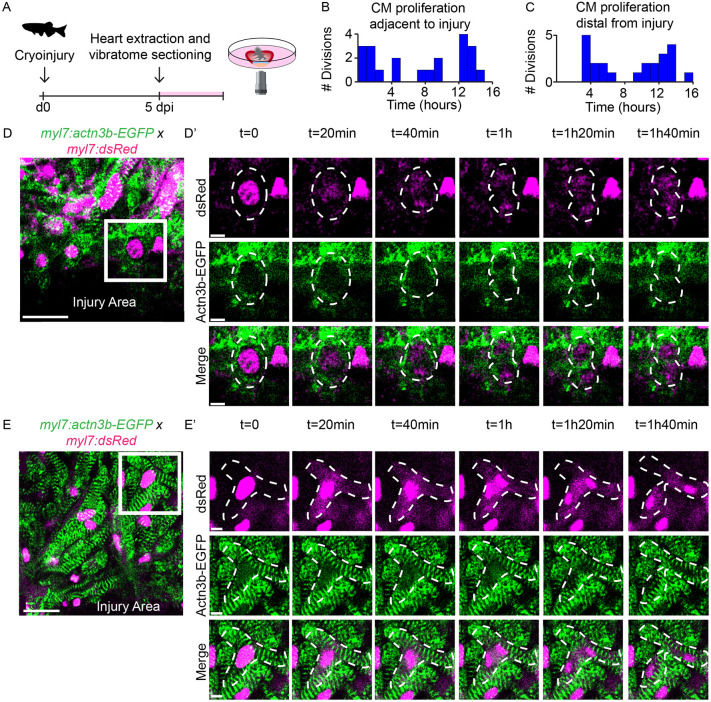
**Dynamics of sarcomere structures during cardiomyocyte proliferation.** (A) Schematic representation of workflow. (B,C) Histogram showing the distribution of proliferation during time-lapse imaging in cardiomyocytes directly adjacent to the injury (B) or more distal to the injury (C). (D-E′) Overview images (D,E) and zoom-ins (D′,E′) of time-lapse imaging on 5 dpi Tg(*myl7:*actn3b-EGFP; *myl7*:DsRed) cardiac slices capturing cardiomyocyte proliferation directly adjacent to the injury (D,D′) or distal to the injury (E,E′). Cardiomyocyte nuclei marked by dsRed are shown in magenta; sarcomeric structures marked by Actn3b-EGFP are shown in green. Dotted lines indicate cellular outlines. Scale bars: 20 µm (overview); 5 µm (zoom).

Sarcomeres form the contractile apparatus of cardiomyocytes and are highly organized in adult myocardial tissue, which is required for efficient contraction. However, sarcomeres may form a barrier for cell divisions (Engel et al., 2006; Williams et al., 2015) and are therefore disassembled before/during cell division and re-assembled when cell division has completed (Ahuja et al., 2004; Fan et al., 2015; Uribe et al., 2018). Sarcomere dynamics during cardiomyocyte proliferation have been studied in the context of the embryonic or neonatal heart when sarcomeres are still very immature. Disassembled sarcomeres are also found in border zone cardiomyocytes of the regenerating zebrafish heart (Jopling et al., 2010); however, very little is known about their dynamics due to a lack of appropriate live imaging tools. To investigate sarcomere dynamics during heart regeneration, we performed live imaging on cardiac slices of 5 dpi hearts of a Tg(*myl7:actn3b-EGFP; myl7:DsRed*), which expresses an actinin-GFP fusion protein that marks the Z-disk and a nuclear-localized dsRed in all cardiomyocytes (Fig. 3A) (Lin et al., 2012). We observed proliferating cardiomyocytes throughout time-lapse imaging (Fig. 3B,C). Strikingly, cardiomyocytes employed very different sarcomere dynamics depending on their location. In cardiomyocytes directly adjacent to the injury, Actn3b-EGFP is very diffuse and localized close to the plasma membrane (Fig. 3D,D′, t=0 and Movie 5). The immature sarcomeres remain disassembled during nuclear division (t=0-40 min), after which the plasma membrane constricts and cleaves the mother cell into two daughter cells (t=1 h-1 h20 min). The Actn3b-EGFP signal in the constricting plasma membrane afterwards slowly diffuses (t=1 h40 min), which might indicate the reassembly of immature, punctae-like sarcomere structures in the new daughter cells. Although in the embryonic heart sarcomere structures disassemble and reassemble to form mature sarcomeres during cell division (Uribe et al., 2018), the sarcomeres in border zone cells remain largely disassembled hours after division, indicating that these processes may be regulated differently.

Strikingly, we found that cardiomyocytes more distal to the injury employ a vastly different method of proliferation, as these cardiomyocytes retain sarcomere structures throughout the whole cell cycle (Fig. 3E,E′ and Movie 6). At t=0, the nucleus is surrounded by sarcomeres. These sarcomeres are locally broken down as the nuclear envelope breaks down, the chromosomes condense and align prior to division (Fig. 3E′, t=0-40 min). Subsequently the nucleus divides, which is followed by reassembly of sarcomeres (t=1h-1h40 min). To simultaneously visualize the sarcomeres and cell membrane, we also imaged cardiac slices prepared from Tg(*myl7:BFP-CAAX;myl7:actn3b-eGFP*) fish (Fig. S4 and Movie 7). From this, we conclude that sarcomere disassembly and reassembly is followed by cytokinesis. Interestingly, the membrane of the dividing cells remains in close contact to the neighboring cardiomyocytes, indicating they remain coupled throughout mitosis.

From these observations, we conclude that cardiomyocytes in the adult heart have at least two different mechanisms for organizing sarcomere structures during cell division. First, cardiomyocytes can have completely disassembled sarcomeres before, during and after cell division. Second, cardiomyocytes can transiently and partially disassemble and reassemble sarcomeres during cell division. Although we observed that the first mechanism occurred mostly in cardiomyocytes located proximal to the wound, the second mechanism was more often observed in cardiomyocytes more distal to the wound. The latter may be the preferred mechanisms for cardiomyocyte proliferation in an uninjured context and could be important to maintain cardiac function during cell division, as these cardiomyocytes remain connected to the neighboring cardiomyocytes.

### The proteasome and calpain inhibitor MG-132 blocks sarcomere disassembly and proliferation

**Fig. 4.**
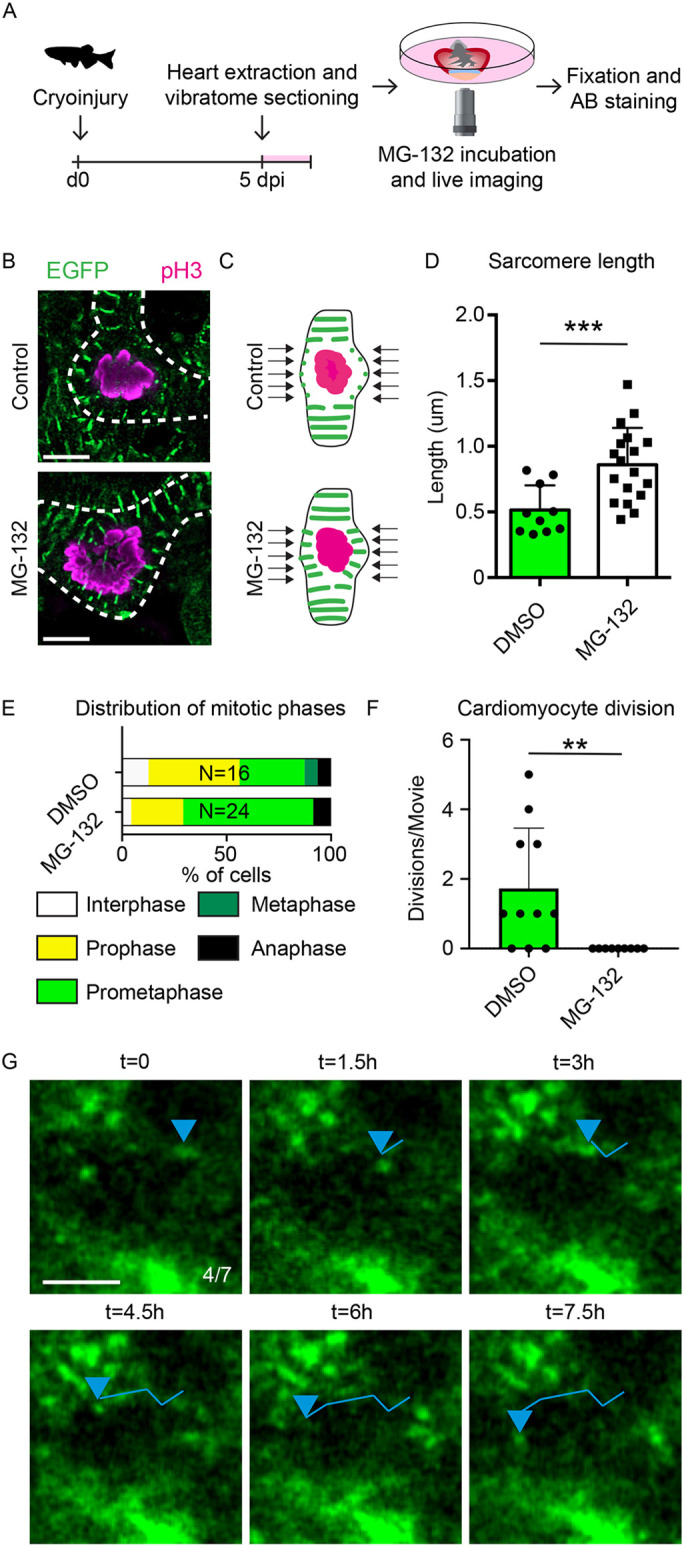
**The proteasome and calpain inhibitor MG-132 blocks sarcomere disassembly and proliferation.** (A) Schematic of experiments in B-H. (B) Confocal images of prometaphase cells from control and MG-132-treated cardiac slices. Mitotic nuclei are marked with pH3 (magenta) and sarcomeres are marked with Actn3b-EGFP (green). Dotted lines represent cellular outlines. Scale bar: 5 µm. (C) Schematic representation of sarcomere length quantification. The length of sarcomeres around the nucleus, indicated by arrows, was measured for quantification. (D) Quantification of sarcomere length surrounding prometaphase nuclei. Each dot represents a result from a single cell. Data are mean±s.d. (E) Distribution of mitotic phases in pH3^+^ cells in cardiac slices. (F) Quantification of cardiomyocyte divisions per time-lapse movie in the presence of DMSO or MG-132. Each dot represents a result from one time-lapse movie. Data are mean±s.d. (G) Tracking of sarcomere fragments in a cardiomyocyte directly adjacent to the injury during MG-132 treatment. Positions of sarcomere fragment are indicated by a blue arrowheads. Tracks are shown by lines. Scale bar: 5 µm. **P<0.01. ***P<0.001.

Cardiomyocyte proliferation was accompanied by highly dynamic sarcomeres surrounding the dividing nucleus (Fig. 3E,E′). Hence, we hypothesized that quick and localized degradation of sarcomeres would be pivotal for proper cardiomyocyte proliferation. Calpains and the proteasome work together during sarcomere degradation, as calpains release sarcomere fragments (Ono and Sorimachi, 2012), after which the individual components become accessible for further proteolysis by the proteasome (Portbury et al., 2011). To test the role of calpains and the proteasome in sarcomere breakdown during cardiomyocyte proliferation, we treated 5 dpi Tg(*myl7:actn3b-EGFP*) cardiac slices with the calpain and proteasome inhibitor MG-132 (Tsubuki et al., 1996) and performed live imaging on these slices (Fig. 4A). Afterwards, these cardiac slices were stained with an anti-phosphorylated histone 3 antibody to label mitotic cells. As our timelapse movies showed very localized disassembly of sarcomeres in close proximity to the mitotic nucleus during proliferation, we quantified the length of these sarcomeres (Fig. 4B,C). Interestingly, we observed that sarcomeres surrounding prometaphase nuclei were significantly larger and therefore more assembled in MG-132-treated cardiac slices compared with controls (Fig. 4B-D). This observation is in accordance with previous observations that ubiquitin-mediated sarcomere degradation occurs around the nucleus during mitosis (Ahuja et al., 2004). Furthermore, quantification of mitotic phases in MG-132 and control-treated cardiac slices revealed that prometaphase cells were over-represented upon proteasome inhibition (Fig. 4E). Altogether, these data point to a model in which ubiquitin-mediated sarcomere breakdown around the nucleus allows prometaphase cells to progress through mitosis.

Following up on this, we analyzed time-lapse imaging performed on these slices and quantified the number of proliferative events. Corroborating an essential role for calpains and the proteasome, cardiomyocyte divisions were no longer observed in the cardiac slices treated with MG-132 (Fig. 4F). Future studies are required to investigate whether impaired cell cycle progression is the result of persisting sarcomeres or of other factors, such as a block in cyclin B proteolysis or induction of apoptosis (Tokumoto et al., 1997; Potapova et al., 2006; Yuan et al., 2008; Guo and Peng, 2013; Dang et al., 2014). Additionally, live imaging on Tg(*myl7:actn3b-EGFP*)-expressing cardiac slices in the presence of MG-132 revealed highly motile α-actinin fragments in cardiomyocytes (Fig. 4G). These motile fragments appeared along the plasma membrane of cardiomyocytes proximal to the injury (Fig. 4G, Movie 8). We never observed these highly motile actinin fragments in DMSO-treated cardiac slices (0/13), suggesting that they resembled (partially) disassembled sarcomeres that are not broken down. Together, these results indicate a pivotal role for calpains and the proteasome in sarcomere disassembly during adult cardiomyocyte proliferation. In addition, these highlight an advantage of cardiac slice cultures, which is the possibility to pharmacologically inhibit or stimulate processes with small molecules that would cause adverse side effects or mortality when provided *in vivo.*

In conclusion, we have presented a cardiac slice-culturing method that allows the temporal monitoring of cellular processes during cardiomyocyte proliferation in the regenerating adult zebrafish heart. In addition, combining cardiac slice cultures with time-lapse imaging and pharmacological treatments provides new insights into sarcomere dynamics during adult cardiomyocyte proliferation. In conjunction with the wide range of transgenic lines available in zebrafish, cardiac slice culture is a valuable tool for future studies into the dynamics underlying cardiomyocyte proliferation in the regenerating zebrafish heart.

## MATERIALS AND METHODS

### Transgenic zebrafish lines and cryoinjury

All procedures involving animals were approved by the local animal experiment committees and performed in compliance with animal welfare laws, guidelines and policies, according to national and European law. Fish housing and husbandry was performed as described previously (Aleström et al., 2020). Fish of both sexes from the following fish lines were used: TL, Tg*(myl7:BFP-CAAX*) (Guerra et al., 2018), TgBAC(nppa:mCitrine) (Honkoop et al., 2019), Tg(EF1α:mAG-hGem(1/60))^rw0412a^ (Sugiyama et al., 2009), Tg(*myl7*:*nucDsRed*) (Rottbauer et al., 2002) and Tg(*myl7:actn3b-EGFP*) (Lin et al., 2012).

Cryoinjury was performed on 4- to 12-month-old zebrafish as previously described (González-Rosa and Mercader, 2012). Animals were excluded from the study in case of signs of aberrant behavior/sickness/infection, according to animal guidelines.

### Cardiac slice culture

All experimental procedures were performed at 28°C to prevent damage to heart tissue resulting from cold shock. Four- to 12-month-old zebrafish were euthanized using an overdose of MS-222 (250 mg/l), before heart extraction in Leibovitz's L-15 medium pre-supplemented with Glutamax (Gibco) and heparin (1 mg/50 ml, Sigma-Aldrich). After removal of the atrium and bulbus arteriosus, hearts were allowed to bleed out for 2 min after which the heartbeat was stopped by keeping hearts in L-15+Glutamax+20 mM BDM (Sigma-Aldrich) for 5 min. Hearts were then embedded in 1% agarose (L-15+Glutamax+20 mM BDM+10%FBS) and kept at 43°C before mounting. Hearts were oriented in agarose with the apex facing down, the former site of the bulbus facing top left and the former site of the atrium facing top right. It is important to orient hearts as quickly and accurately as possible to prevent hearts from popping out during vibratome sectioning. After orientation, agarose blocks were allowed to set for 5 min and then sectioned using a vibratome [Microm HM650V, Thermo Scientific, Trim=200 µm, v=10 (1 mm/s), amplitude=1.0 mm, frequency=85 Hz] containing L-15+Glutamax and 20 mM BDM. Sections were collected in fresh L-15+Glutamax and 20 mM BDM. Surrounding agarose was subsequently carefully removed and sections were transferred to 5 ml culture medium [L-15+Glutmax+20 mM BDM+10% FBS+100 µg/ml Primocin (Invitrogen)+1% Pen/Strep] in a six-well plate and kept at 28°C in ambient oxygen conditions. For 3 day culture experiments, culture medium was exchanged on day 2.

### Time-lapse imaging

Cardiac slices were transferred to a four-chamber glass-bottomed dish (Greiner), residual medium was removed and sections were embedded in 1% agarose (L-15+Glutamax+20 mM BDM+10%FBS). Agarose was allowed to set for 1 min before culture medium (1 ml per chamber) was added and confocal imaging was performed at 28°C. Imaging on Tg(*EF1α*:mAg-hGem) cardiac slices was performed on a SP5 confocal microscope (Leica). *Z*-stacks with a *z*-step size of 2µm were acquired every 45 min using a 20× objective for 16 h. Imaging on Tg(*myl7:BFP-CAAX; myl7:dsRed*) and Tg(*myl7:BFP-CAAX; myl7:actn3b-EGFP*) cardiac slices was performed on a SP8MP confocal microscope (Leica). BFP was excited using a multiphoton laser at a wavelength of 800 nm; GFP and dsRed were imaged using single-photon confocal microscopy. *Z*-stacks with a *z*-step size of 1 µm were acquired every 15 min using a 20× objective with 3× digital zoom. Imaging on Tg(*myl7*:*actn3b-EGFP; myl7:dsRed*) cardiac slices was performed on a SP5 and SP8MP confocal microscope (Leica). *Z*-stacks with a *z*-step size of 1 µm were acquired every 10 min using a 20× objective with 3× digital zoom (for proliferation counting) or 5× digital zoom (for analysis of sarcomeric fragments after MG132 treatment).

MG-132 (Sigma-Aldrich) treatment was performed at a concentration of 20 µM in culture medium. Pharmacological treatment was initiated directly after embedding of cardiac slices in glass bottom dishes and persisted during time-lapse imaging.

### Immunofluorescent stainings

Vibratome slices or whole hearts were subsequently fixed in 4% PFA, equilibrated in 30% sucrose, embedded in OCT tissue-freezing medium and sectioned into 10 µm sections in a cryostat. Cryosections were equally distributed onto serial slides so each slide contained sections representing all areas of the ventricle or cardiac slice.

Primary antibodies used were: polyclonal rabbit-anti-MEF2c (Biorbyt, #orb256682, 1:1000 or Abcam, #ab197070, 1:500), monoclonal mouse-anti-PCNA (DAKO #M0879, 1:800), monoclonal mouse-anti-N2.261 (DSHB, #AB_531790, 1:200), chicken-anti-GFP (Aves #GFP-1010, 1:500) and rabbit-anti-pH3 (Ser10) (Millipore, #06-570, 1:100). Antigen retrieval was performed by heating slides containing heart sections at 85°C in 10 mM sodium citrate buffer (pH 6) for 10 min. Secondary antibodies conjugated to Alexa 488, Alexa 555 (ThermoFisher Scientific) or Cy5 (Jackson Laboratories) were used at a 1:500 dilution. Nuclei were shown by DAPI staining. Immunofluorescent stainings were imaged using a VS200 slide scanner (Olympus). TUNEL staining was performed using a In Situ Cell Death Detection Kit, Fluorescein (Sigma Aldrich) during secondary antibody incubation.

### Quantification of proliferative events

All quantifications in this manuscript were carried out blinded. All image analysis was performed in Imaris (Bitplane). Quantification of MEF2/PCNA or was performed by assigning spots to MEF2-positive nuclei and selection of MEF2/PCNA double-positive nuclei. Spots were counted in a region of 200 µm from the injury (border zone) or in squares of 100 µm×100 µm in the remote myocardium (two per section). Two to three sections per heart were counted and the average was used for statistical analysis.

For *myl7*:dsRed proliferation quantification, spots were assigned to all *myl7*:dsRed-positive nuclei and the percentage of nuclei that underwent cytokinesis during the time course of the experiment was calculated by dividing the total amount of nuclei that underwent cytokinesis by the total amount of nuclei at the start of the time-lapse movies.

Proliferative events in the Tg(*myl7*:*actn3b-EGFP*) transgenic line, as described in Fig. 3, were counted for MG-132-treated and control cardiac slices. The incidence of moving sarcomeres was determined per movie where the incidence of a single phenomenon as mentioned in Fig. 4 was considered as a positive case.

### Quantification of sarcomere length

Quantification of sarcomere length was performed on images acquired on a LSM900 airyscan2 confocal microscope (ZEISS). Acquired images were loaded into FIJI. Length of sarcomeres surrounding prometaphase nuclei were measured in a single *z*-plane precisely in the center of the nucleus. Sarcomeres were quantified only when their orientation was directed towards the nucleus. Sarcomeres that were more peripheral to the cardiomyocyte were not taken into account for this quantification. The average length of sarcomeres in one cell was used for statistical testing.

### Quantification of mitotic phase

For quantification of mitotic phases in cardiac slices treated by MG-132 and control slices, all pH3^+^ cells in stained cryosections were imaged using a SPE confocal microscope (Leica). These cells were then classified for mitotic phase based on nuclear morphology of the pH3^+^ cells (Tang et al., 2012).

### Timing and location of mitotic exit and proliferation

Timing of proliferation and mitotic exit was quantified from 16 h time-lapse movies. Mitotic exit was defined as the timepoint when increasing signal in Tg(*EF1α*:mAg-hGem) cardiac slices sharply declined. Proliferation was defined as the moment when a nucleus splits into two for Tg(*myl7*:*DsRed*) cardiac slices. Proliferation in the Tg(*myl7*:*actn3b-EGFP*) cardiac slices was defined as the moment when the membrane constricts, as described in Fig. 3.

### Statistical analysis

Statistical analysis was performed in Prism. Percentages of MEF2/PCNA-, EdU/MEF2- and TUNEL/DAPI-positive cells, and MEF2 cell density in zebrafish hearts and cardiac slices were compared using a one-way ANOVA. Multiple comparisons were performed and *P*-values were corrected for multiple testing using a Tukey's correction. Statistical analysis for all other experiments was performed using an unpaired *t*-test to test for significance.

### Live imaging in microfluidic chip

Microfluidic experiments were performed as described previously (van Oostrom et al., 2021) with minor adaptions. Briefly, microfluidic chips were generated by casting PDMS (polydimethylsiloxane) in a mold. The chip was designed to have three inlet openings, one outlet opening and a chamber in which tissue can be loaded. This chamber is closed by PDMS pillars, which trap the tissue inside the chamber and prevent entry of air bubbles into the chamber. After curing, the PDMS chip was cut out of the mold and bonded onto a glass slide using a Diener Femto plasma oven. To prevent air bubbles in the chip during imaging, the chip was submersed in sterile PBS supplemented with 1% penicillin/streptavidin for at least 2 h.

Culture medium was loaded onto syringes and degassed in a dessicator together with the chip. Next, the microfluidic chamber was coated with BSA by flushing 2.5% BSA through the outlet tubing at a low flow rate (20 µl/h) for at least 2 h. The chip was then flushed with culture medium, removing remaining air bubbles before loading of tissue onto the microfluidic chamber. Inlet tubing attached to syringes was subsequently attached to the inlets and remaining entries to the microfluidic chip were closed by PDMS-filled tubing. The flow rate was set to 60 µl/h during the experiment. During imaging, the chip was kept in a humidified chamber to prevent air bubbles from entering the chip and inlet tubing.

### Quantification of cell death

Cell death was quantified by two means. First the number of DAPI-positive nuclei was automatically counted using Imaris imaging software in sections. From this, the TUNEL-positive cells were manually selected to determine the ratio of TUNEL^+^/DAPI^+^ cells. A minimum of three cryosections were counted per heart and the average was used for statistical analysis.

Second, the number of nuclei stained by MEF2 were automatically counted using Imaris imaging software to determine MEF2 cell density in sections. Cells were counted in two random squares of 200 µm×200 µm throughout the trabeculated myocardium per cryosections. A minimum of three cryosections were counted per heart and the average was used for statistical analysis.

### EdU incorporation

EdU incorporation experiments were performed using a Click-iT EdU Cell Proliferation Kit for Imaging (ThermoFisher Scientific). In short, EdU was added to culture medium for the desired amount of time (50 μg/ml). After addition of EdU, cardiac slices were placed on ice in the dark for 30 min prior to culture at 28°C in the dark. Cardiac slices were then subsequently fixed in 4% PFA and cryosectioned. Immunofluorescent staining was then performed on the cryosections before Click-iT chemistry was performed following the standard protocol from the manufacturer to stain for incorporated EdU.

## Supplementary Material

Supplementary information

Reviewer comments
