# Supplementary material for: Live imaging of adult zebrafish cardiomyocyte proliferation ex vivo
Source: Development. 2021 Sep 13;148(18):dev199740. doi: 10.1242/dev.199740 (PMC8489017; doi:10.1242/dev.199740)
Supplement: Supplementary information [file develop-148-199740-s1.pdf]

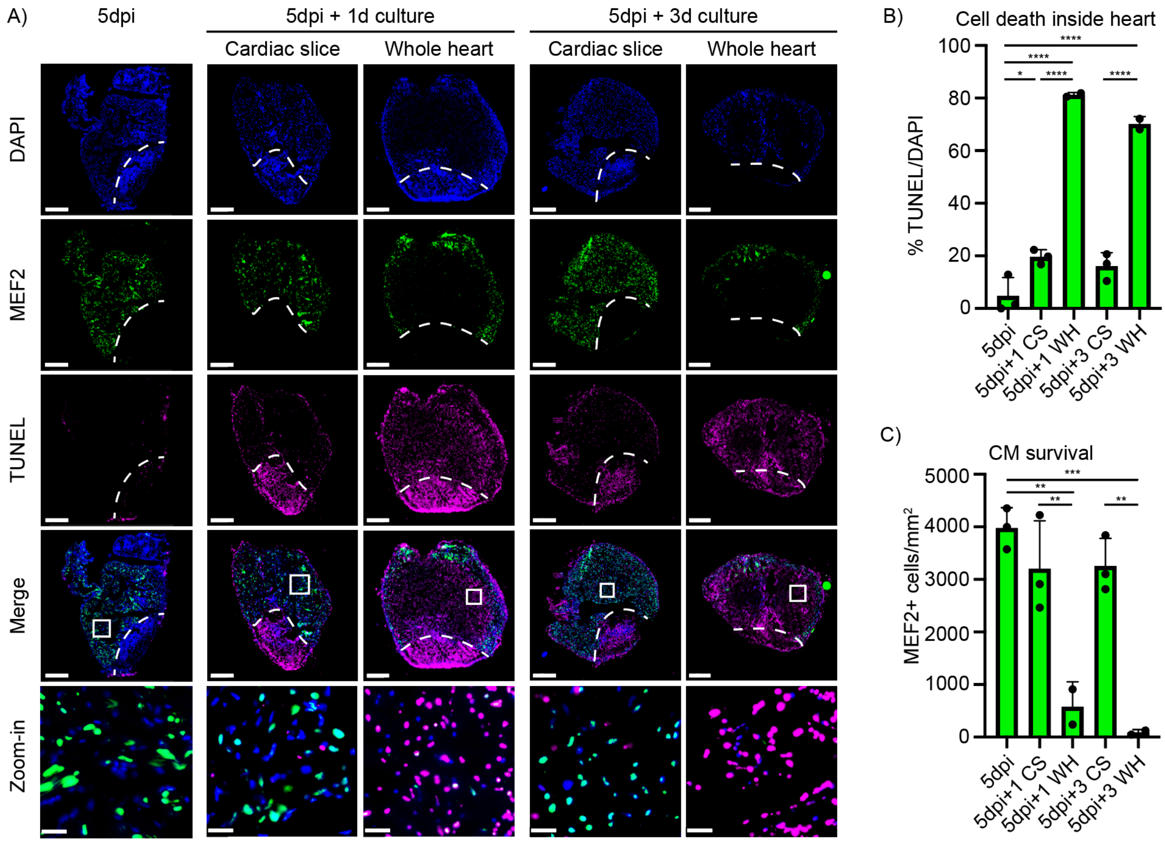

Fig. S1. Cell survival in cardiac slices during culture. (A) Immunofluorescent staining on uncultured hearts, cardiac slices cultures and whole heart cultures. Nuclei are marked by DAPI (blue), cardiomyocyte nuclei by MEF2 (green) and fragmented DNA by TUNEL (magenta). Dotted lines indicate the injury area. Scale bars represent 200µm (overview) or 20 µm (zoom-in). (B) Quantification of cell death in the trabeculated myocardium of 5dpi uncultured hearts, cardiac slice cultures and whole heart cultures.Each dot represents a heart or cardiac slice. Error bars indicate SD. (C) Quantification of cardiomyocyte survival in the trabeculated myocardium of 5dpi uncultured hearts, cardiac slice cultures and whole heart cultures. Each dot represents a heart or cardiac slice. All groups were compared to the 5dpi control situation. Moreover, cardiac slices and whole hearts from the same culture period were compared. All significant differences are shown. Error bars indicate SD. * p-value <0.05, ** p-value <0.01, *** p-value <0.001, **** p-value < 0.0001.

Development • Supplementary information

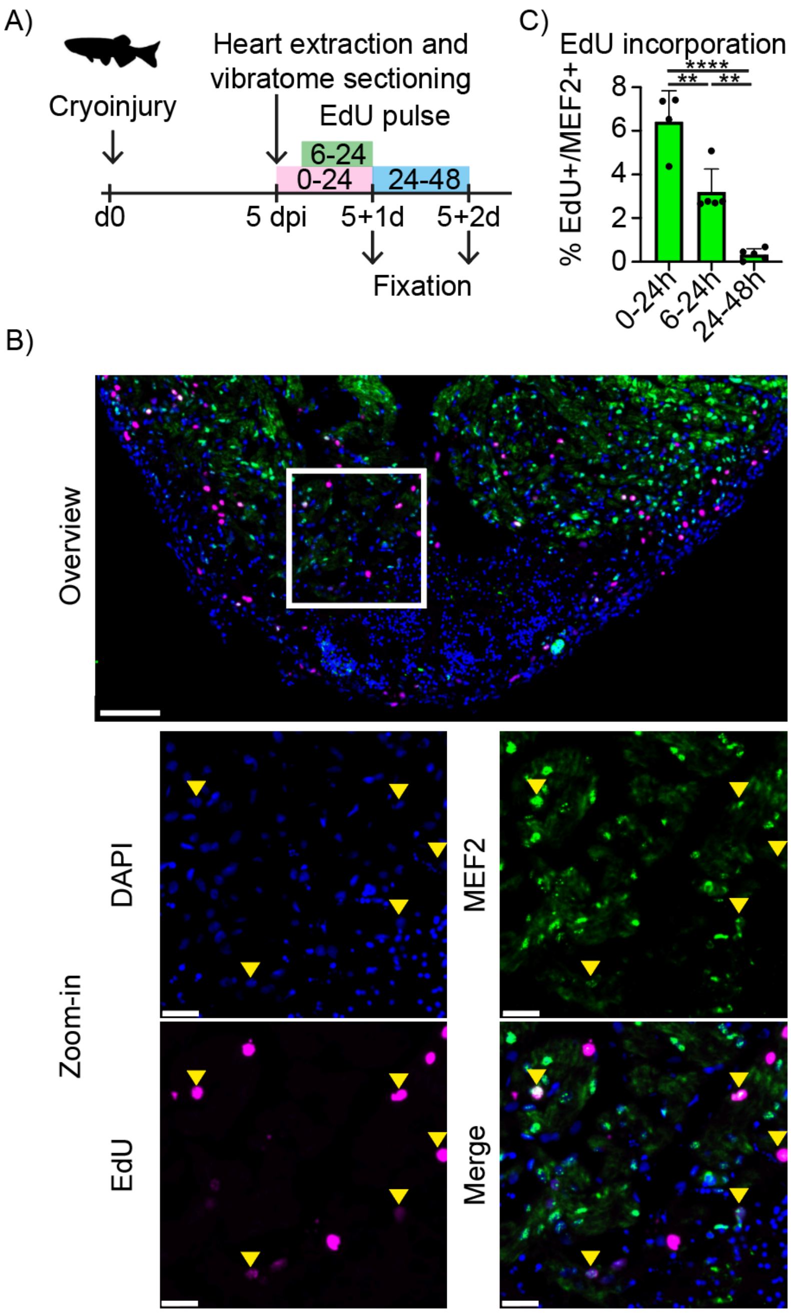

Fig. S2. Cell survival in cardiac slices during culture. (A) Schematic representation of workflow. (B) Immunofluorescent staining on cardiac slice cultures that were pulsed with EdU for the first 24 hours. Nuclei are marked by DAPI (blue), cardiomyocyte nuclei by MEF2 (green) and newly synthesized DNA by EdU (magenta). Yellow arrows in zoom-ins indicate proliferating cardiomyocytes. Scale bars indicate 50µm (overview) or 20µm (zoom-in). (C) Quantification of EdU incorporation in cardiomyocytes during different EdU pulses. Each dot represents a cardiac slice. Error bars indicate SD. ** p-value <0.01, **** p-value < 0.0001.

Development • Supplementary information

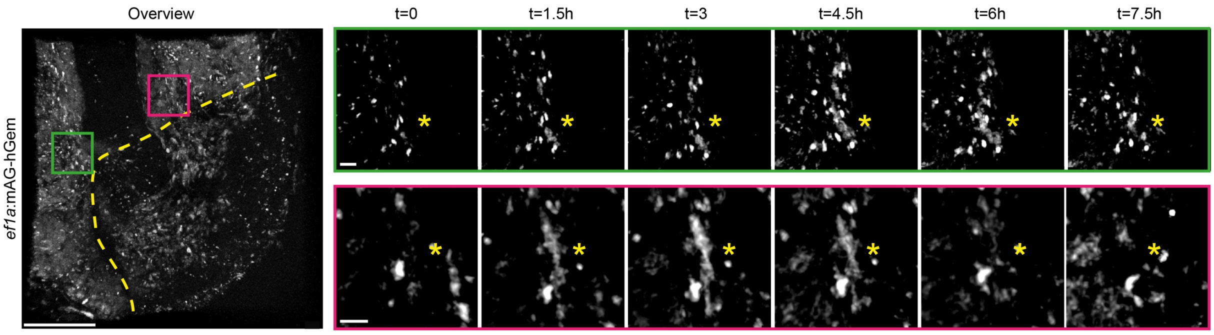

Fig. 3. Live imaging proliferation in a microfluidic chamber. Time-lapse imaging on cardiac slices from 5 dpi *Tg(EF1α:mAg-hGem)* hearts inside a microfluidic chamber, to visualize cells that enter G2/M phase. Yellow dotted line in overview image indicates the injury. Green and magenta squares show regions of zoom-ins. Asterisks in zoom-ins mark cells with dynamic geminin expression. Scalebars represent 200 µm (overview) or 20 µm (zoom).

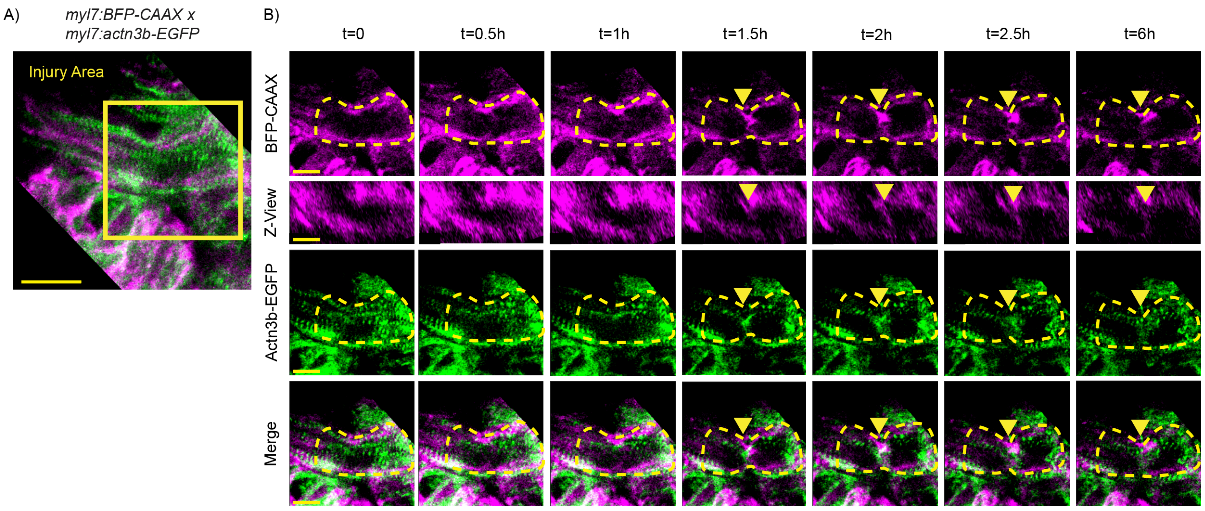

Fig. 4. Sarcomere dynamics during cytokinesis. (A and B) Overview (A) and zoom-ins (B) of time-lapse imaging on cardiac slices from 5 dpi *Tg(myl7:BFP-CAAX;myl7:actn3b-EGFP)* hearts. BFP-CAAX marks membranes (magenta) and Actn3b-EGFP marks sarcomeres (green). Yellow dotted line in zoom-ins indicates cellular outline. Yellow arrows indicate the membrane splitting two daughter cells. Scalebars represent 20 µm or 10µm (zoom in).

Development • Supplementary information

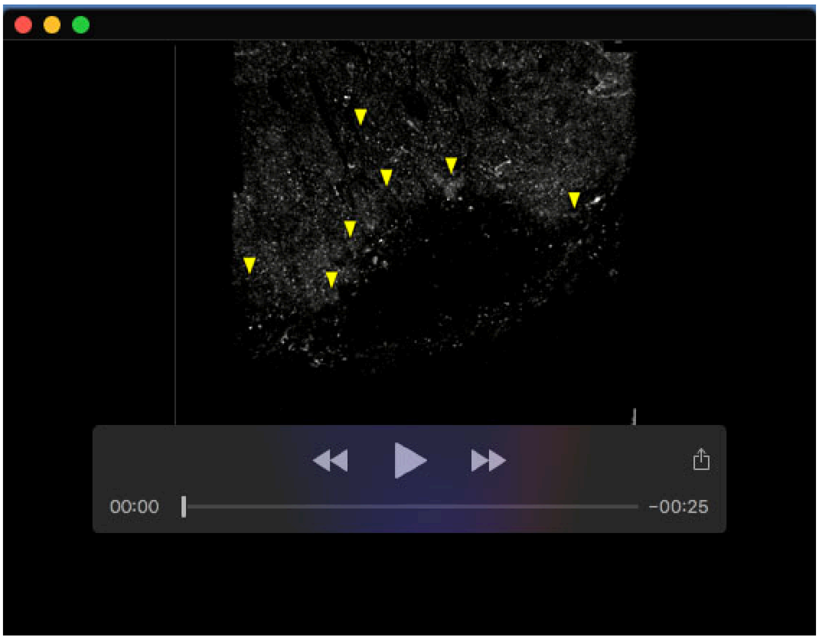

Movie 1. Time-lapse imaging of injured Tg(*EF1α*:mAg-hGem) zebrafish heart (related to Figure 2B). Arrows indicate proliferative events. Each frame represents 45 minutes.

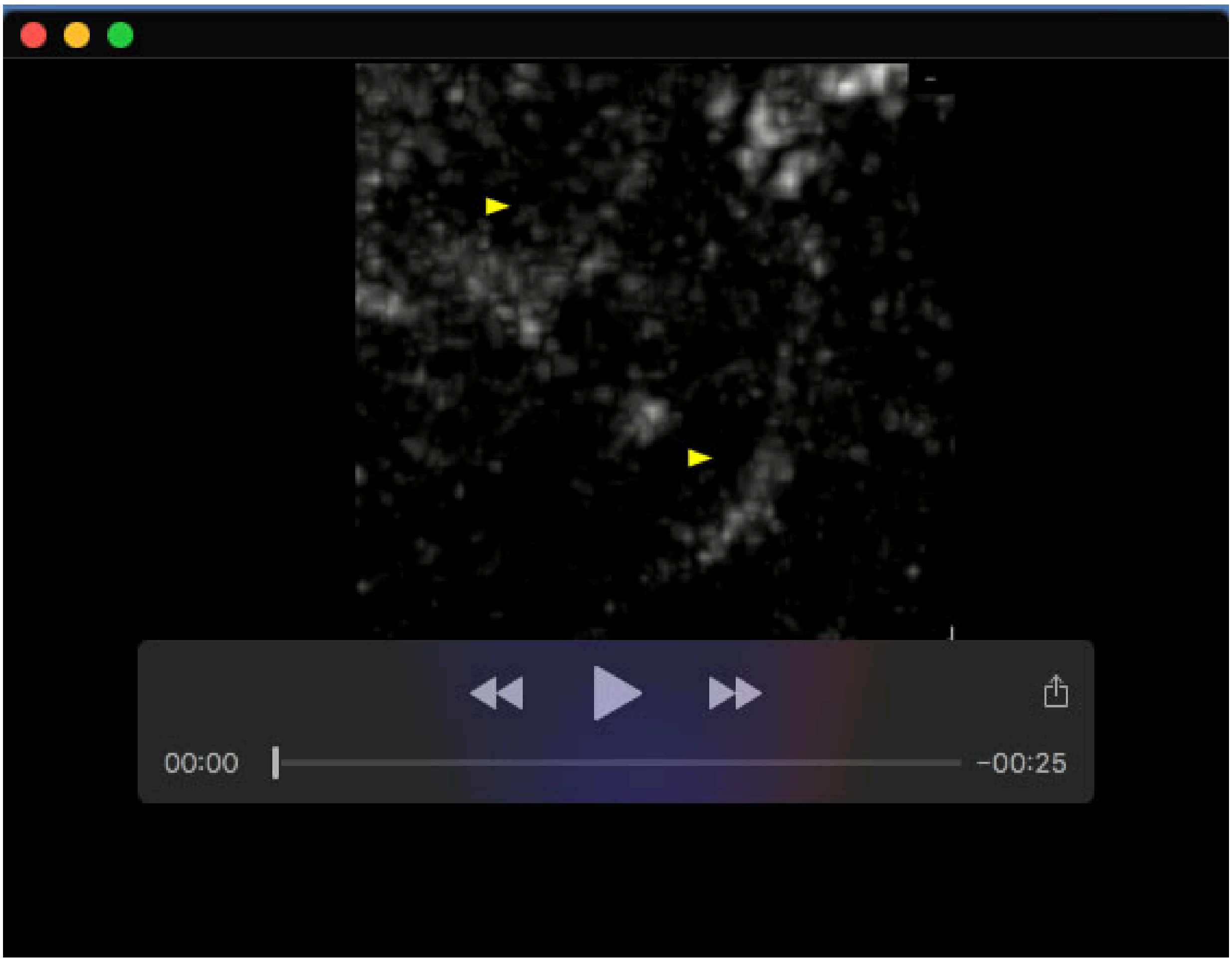

Movie 2. Zoom-in of red region of time-lapse imaging of injured Tg(*EF1α*:mAg-hGem) zebrafish heart (related to Figure 2B'). Arrows indicate proliferative events. Each frame represents 45 minutes.

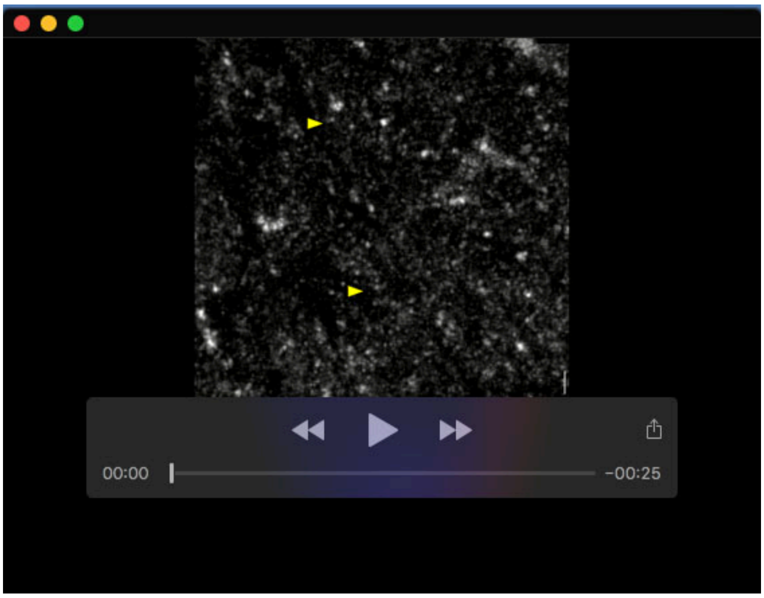

Movie 3. Zoom-in of blue region of time-lapse imaging of injured Tg(*EF1α*:mAg-hGem) zebrafish heart (related to Figure 2B'). Arrows indicate proliferative events. Each frame represents 45 minutes.

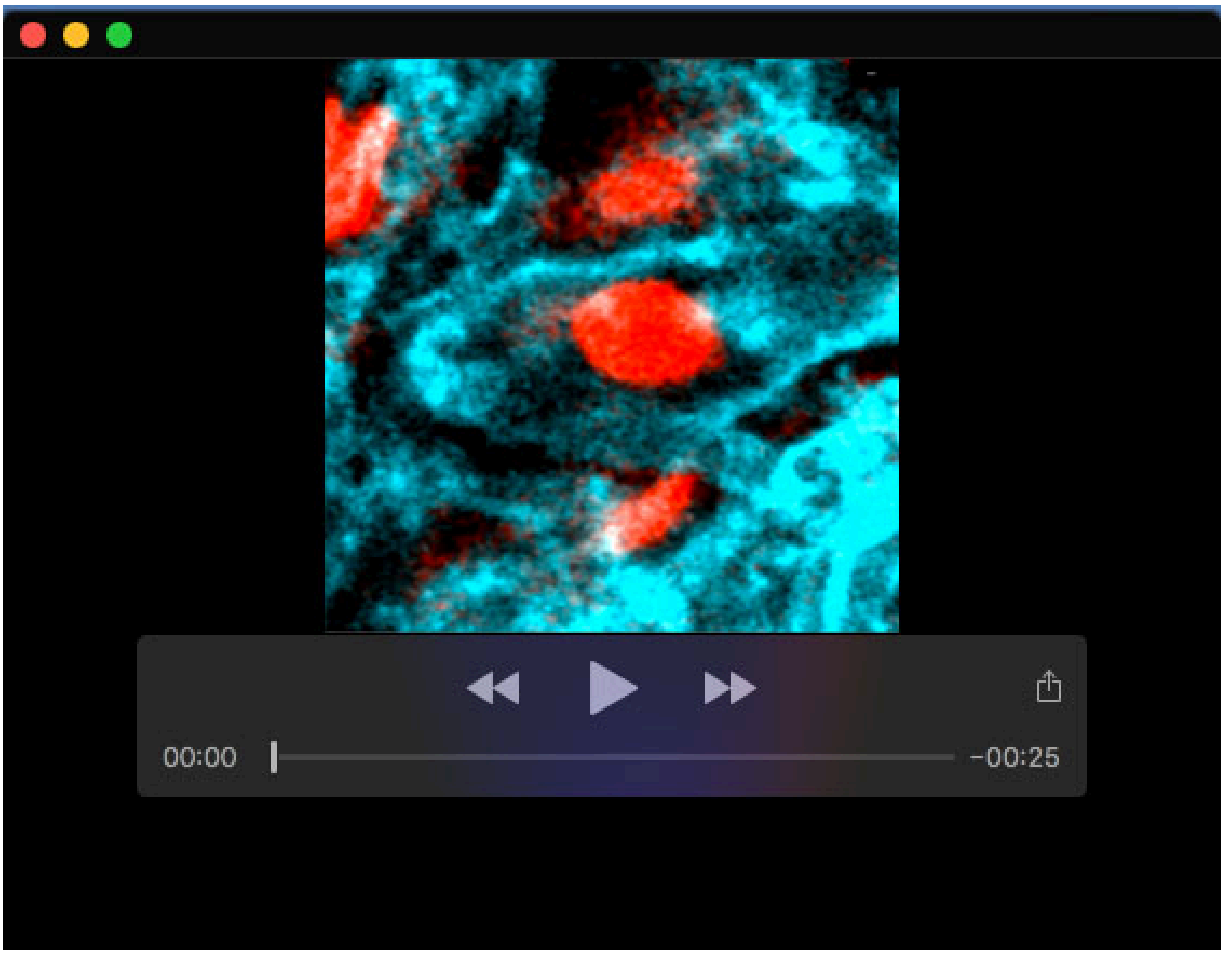

Movie 4. Time-lapse imaging of nuclear division and cytokinesis in cardiomyocytes in a Tg(*myl7:dsRed;myl7:BFP-CAAX)* 5dpi zebrafish heart (related to Figure 2F'). Cardiomyocyte nuclei are shown in red, cardiomyocyte membranes are shown in cyan. Each frame represents 15 minutes.

Development • Supplementary information

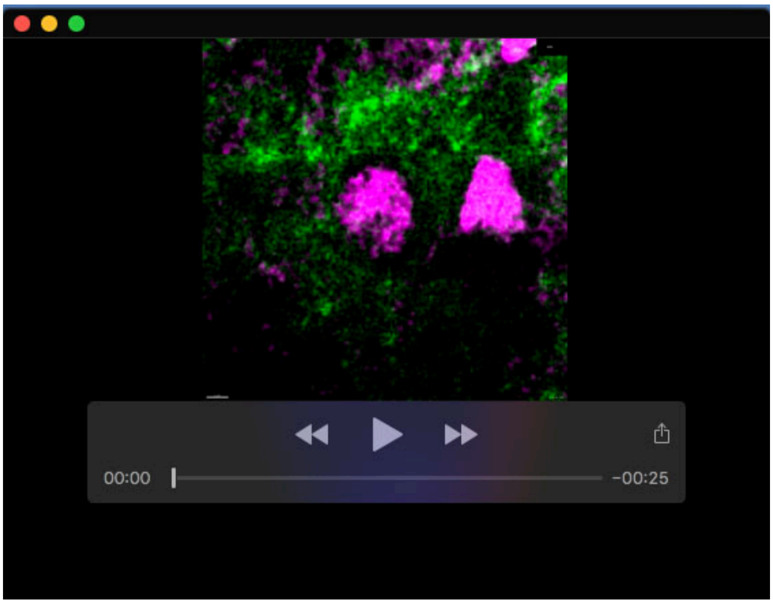

Movie 5. Time-lapse imaging of proliferating cardiomyocyte directly adjacent to the injury in a Tg(*myl7:dsRed;myl7*:*actn3b-EGFP)* 5dpi zebrafish heart (related to Figure 3D'). Cardiomyocyte nuclei are shown in magenta and sarcomeres are shown in green. Each frame represents 10 minutes.

Development • Supplementary information

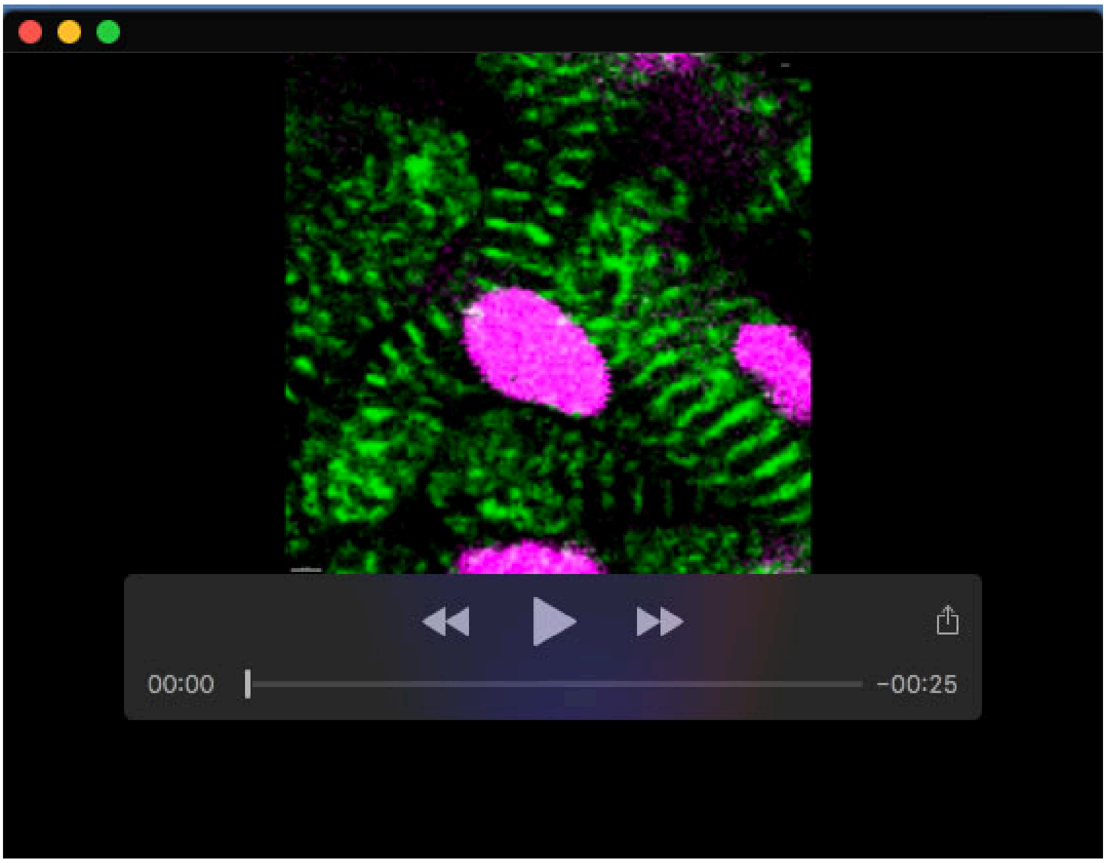

Movie 6. Time-lapse imaging of proliferating cardiomyocyte more distal from the injury in a Tg(*myl7:dsRed;myl7:actn3b-EGFP)* 5dpi zebrafish heart (related to Figure 3E'). Cardiomyocyte nuclei are shown in magenta and sarcomeres are shown in green. Each frame represents 10 minutes.

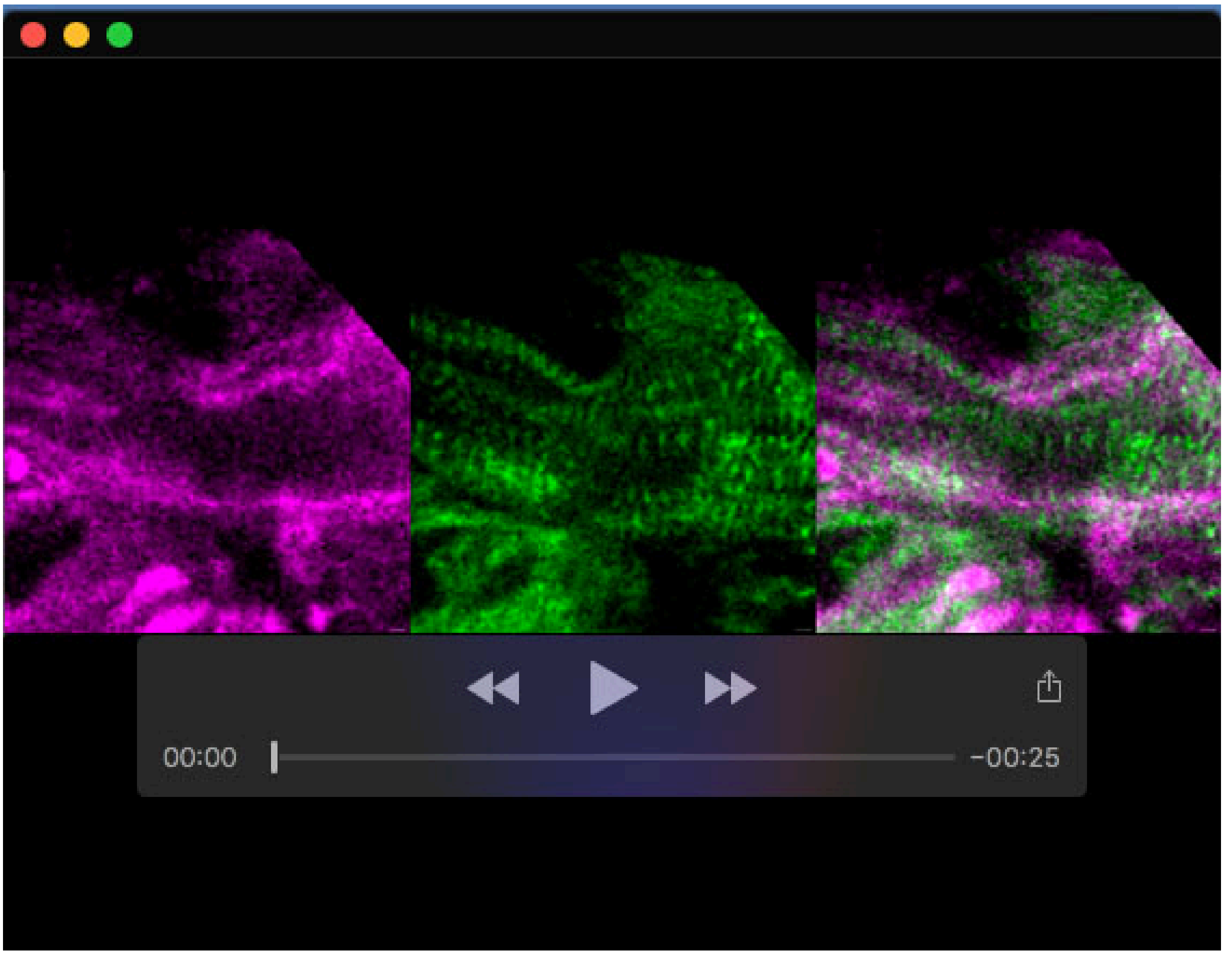

Movie 7. Time-lapse imaging of proliferating cardiomyocyte in a Tg(*myl7:BFP-CAAX;myl7:actn3b-EGFP)* 5dpi zebrafish heart (related to Supplementary Figure 4). Cardiomyocyte membranes are shown in magenta and sarcomeres are shown in green. Each frame represents 15 minutes.

Development • Supplementary information

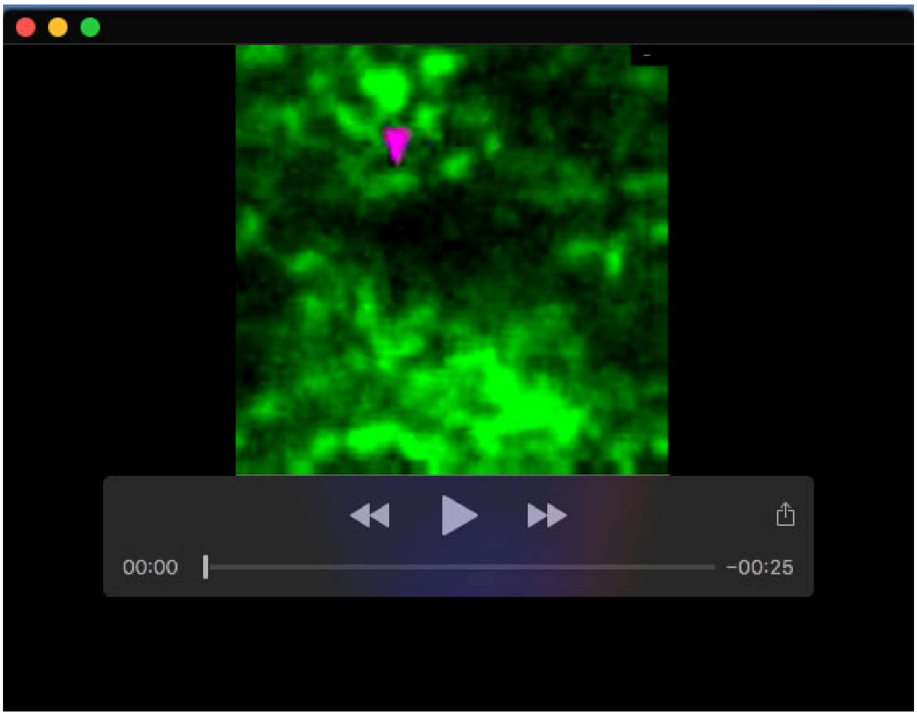

Movie 8. Time-lapse imaging of sarcomere fragments in a cardiomyocyte directly adjacent to the injury in a Tg(*myl7*:Actn3b-EGFP) zebrafish heart (related to Figure 4G). Colored arrows represent sarcomere fragments in Figure 4G. Each frame represents 30 minutes.

Development • Supplementary information
