# Supplementary material for: Live imaging of adult zebrafish cardiomyocyte proliferation ex vivo
Source: Development. 2021 Sep 13;148(18):dev199740. doi: 10.1242/dev.199740 (PMC8489017; doi:10.1242/dev.199740)
Supplement: Reviewer comments [file develop-148-199740_review_history.pdf]

Live imaging of adult zebrafish cardiomyocyte proliferation *ex vivo*

Hessel Honkoop, Phong D. Nguyen, Veronique E. M. van der Velden, Katharina F. Sonnen and Jeroen Bakkers

DOI: 10.1242/dev.199740

Editor: Kenneth Poss

Review timeline

Original submission:	23 April 2021
Editorial decision:	17 May 2021
First revision received:	20 July 2021
Accepted:	4 August 2021

Original submission

First decision letter

MS ID#: DEVELOP/2021/199740

MS TITLE: Live imaging of adult zebrafish cardiomyocyte proliferation ex vivo

AUTHORS: Hessel Honkoop, Phong D Nguyen, Veronique E.M. van der Velden, Katharina F Sonnen, and Jeroen Bakkers

I have now received all the referees' reports on the above manuscript, and have reached a decision. The referees' comments are appended below, or you can access them online: please go to BenchPress and click on the 'Manuscripts with Decisions' queue in the Author Area.

As you will see, the referees express considerable interest in your work but have some significant criticisms and recommend a substantial revision of your manuscript before we can consider publication. If you are able to revise the manuscript along the lines suggested, which likely involves further experiments - e.g. the use of more targeted inhibitors of cardiomyocyte proliferation per Review 2 and other reviewer comments, I will be happy to receive a revised version of the manuscript. Your revised paper will be re-reviewed by one or more of the original referees, and acceptance of your manuscript will depend on your addressing satisfactorily the reviewers' major concerns. Please also note that Development will normally permit only one round of major revision.

We are aware that you may be experiencing disruption to the normal running of your lab that make experimental revisions challenging. If it would be helpful, we encourage you to contact us to discuss your revision in greater detail. Please send us a point-by-point response indicating where you are able to address concerns raised (either experimentally or by changes to the text) and where you will not be able to do so within the normal timeframe of a revision. We will then provide further guidance. Please also note that we are happy to extend revision timeframes as necessary.

Please attend to all of the reviewers' comments and ensure that you clearly highlight all changes made in the revised manuscript. Please avoid using 'Tracked changes' in Word files as these are lost in PDF conversion. I should be grateful if you would also provide a point-by-point response detailing how you have dealt with the points raised by the reviewers in the 'Response to Reviewers' box. If you do not agree with any of their criticisms or suggestions please explain clearly why this is so.

© 2021. Published by The Company of Biologists under the terms of the Creative Commons Attribution License (https://creativecommons.org/licenses/by/4.0/).

Reviewer 1

Advance summary and potential significance to field

Honkoop et al developed an ex vivo culture system for zebrafish cardiac slices that allows for timelapse imaging of and delicate chemical perturbation of regenerating myocardium. This system has the potential to improve ex vivo screens of zebrafish cardiomyocytes in response to various injury, mechanical, and chemical conditions; while avoiding the necrotic complications of some earlier ex vivo systems.

Comments for the author

We here raise a few minor comments that may clarify the paper for readers before acceptance.
Minor Comments:

1) The authors note that the cardiac slices can be cultured ex vivo for at most three days. They hypothesize that the protocol could be modified to extend the longevity of the culture but do not conduct any further experiments to assess these hypotheses. The authors may want to consider culturing the slices with an air liquid interface (growing them in a trans well) to extend the culture longevity.

2) In Fig 1B, using the color cyan overlapping with the yellow mCitrine produces a white color that does not stand out dramatically from yellow. Instead of cyan, can another color be used that "pops out" more from the yellow mCitrine background in the merged panels?

3) In Fig 2B, arrows indicating border zone vs remote zone could be helpful to the reader - also, in 2F' is there a way to enhance the magenta signal to increase its contrast when overlapped with green?

4) Units should be indicated in the vibratome velocity in “Cardiac slice culture” methods section.

5) MG-132 also initiates apoptosis. Please discuss how this may contribute to the decrease in cardiomyocyte divisions observed.

6) Please specify what "frequency" is in Figures 2 and 3.

Reviewer 2

Advance summary and potential significance to field

In this manuscript, Honkoop et al. developed a cardiac slice culture system to monitor myocardium regeneration after heart injury. The system maintains CM proliferation for 2 days and is adaptable for live imaging and chemical manipulations. The authors further showed an application of the system by showing that proteasome and calpain inhibition blocks sarcomere disassembly and proliferation. The new system is potentially of high interest to the field. However, the study is largely preliminary and has issues with data quality. It does not provide novel information.

Comments for the author

Major:
1. The authors failed to demonstrate applications of the system. It is of no surprise that MG132 induces cell cycle arrest. Known signaling pathways that regulate heart regeneration should be tested instead, for instance, by inhibiting the Fgf pathway or treating with Alfacalcidol.

2. BDM is a myosin II inhibitor that may arrest the cell cycle and block cytokinesis. BDM inhibits contraction of the contractile ring of the cleavage furrow during cytokinesis. It is unclear how the system mimics the in vivo situation and whether the claimed cytokinesis is reliable.

© 2021. Published by The Company of Biologists under the terms of the Creative Commons Attribution License (https://creativecommons.org/licenses/by/4.0/).

3. CM proliferation was primarily observed in the first 2 days of culture. These division events are likely ongoing proliferation at the time of heart isolation. It is possible that no new CM proliferation was initiated during culture. If true, it reduces the value of the system.

4. Most videos and images are blurry. A time interval of 30-45 min is way too long. A shorter window of 5-10 min is necessary to visualize cellular details of division. The evidence of cytokinesis is not promising. The dynamics of sarcomere structure is potentially a novel discovery; however, the claims are not supported by the blurry images and arbitrary outlines and quantifications. In Figure 4H and Movie 10, the temporal resolution does not allow me to track these fragments.

5. The authors' claim that the "use of PCNA as a marker for cardiomyocyte proliferation may result in an overestimation of the actual number of cell divisions" is questionable. PCNA expression increases from the late G1 phase through the S-phase of the cell cycle. Theoretically, PCNA staining marks proliferating cells from the late G1 to M phase and labels more cells than the G2/M phase marker pH3 and the M phase sign of nuclear division. Unless every single proliferating cell was followed from the G1 phase to the M phase, there is no evidence to support this claim in the current study. On the other hand, the authors' observation may suggest that cell cycle progression may be blocked in some CMs (PCNA+ but never divide). The FUCCI reporters or long-term EdU incorporation can be used to clarify this.

6. This study focused on CM proliferation. It would be informative to show whether other cells (i.e., epicardial cells, endocardial cells, endothelial cells) proliferate? Figure S1A. Why were the outflow tract and atrium kept in the 5-dpi culture but not in others? Were these regions counted in B? Were the blood clot regions measured? There are many blood cells in these regions that may affect the conclusion if they are counted.

The authors should separate cell types (e.g., CMs, endocardial cells, epicardial cells). The authors claimed that no necrosis was observed. However, there is no clear data to prove this.
Also, is there apoptosis?

7. Figure 2F'. The schematic of cell shapes is very arbitrary. The cell boundaries are not evident in the video. A membrane maker seems necessary.

8. Figure 3EF. The distal regions in both figure panels contain both well-organized and less-assembled sarcomeres. I cannot draw a clear conclusion from these two images. Again, the outlining of cell boundaries in panel F' is very arbitrary.

9. Figure 3E'. The images do not bear enough resolution to show completed cytokinesis.

10. Figure 4BC. Why were those regions chosen for the quantifications?

Minor:
11. Figure 1D. Quantification of MEF2+/PCNA+ cells was done in the border zone of injured hearts/cardiac slices but in the remote area of uninjured hearts/cardiac slices. Why were different regions counted?

<u>Reviewer 3</u>

Advance summary and potential significance to field

In this manuscript, Honkoop and colleagues describe an adult zebrafish cardiac slice system to study cardiomyocyte proliferation ex vivo. The authors collect adult hearts after cryoinjury, include them in agarose and prepare slices using a vibratome. These cardiac slices can be cultured for a few days with better tissue preservation than other methods that use the whole ventricle. The authors propose this system as a new opportunity to study cell division ex vivo, as they provide evidence that CMs continue to divide in the slices. Importantly, this technique allows them to analyze the image cytokinesis and sarcomere disassembly ex vivo.

© 2021. Published by The Company of Biologists under the terms of the Creative Commons Attribution License (https://creativecommons.org/licenses/by/4.0/).

As noted below, I find that most of the conclusions related to cytokinesis sarcomeric disassembly etc., might be premature and require further analysis using more specific transgenic lines. However, I find that the present manuscript may be of interest to the regeneration community from a methodological perspective. I find that the text is well written, easy to follow, and appropriately referenced.

Comments for the author

Major issues:
(1) Some of the conclusions from the sarcomeric dynamic, nuclear division, etc.,
might be a bit premature. I'm missing some additional tools to support most of these conclusions. For example, although valuable and didactic, the authors have used dashed lines to indicate the border between cells. Inspecting the images -and this might be a limitation of the PDF file provided to the reviewers-, I do not see clear boundaries in most cases. This could be solved using any of the multiple available transgenic lines that express membrane-bound fluorescent proteins in cardiomyocytes. The same is true for some nuclear labelings (see specific examples below). However, I'm not in favor of asking the authors to repeat their experiments with several new transgenes to satisfy this reviewer.
Instead, I would strongly recommend adding a paragraph in the text where the authors acknowledge these limitations and discuss future experiments to tackle these gaps.

Specific examples:
Fig. 2F' — The myl7:DsRed line is helpful to identify cardiomyocyte nuclei, but it is most useful in uninjured hearts. In proliferating cardiomyocytes, the DsRed signaling becomes very diffuse (Lepilina et al., 2006; Wills et al., 2008), and the identification of the nuclei becomes very difficult. This "diffuse" pattern is because the DsRed here has an nls sequence, which directs the protein to the nucleus, and it is not a histone-fused protein. An H2B-XFP would be much more appropriate for this task: otherwise, we're missing mitosis altogether. The use of nppa:Citrine is not particularly useful in this context.

Fig. 3E' and 3E'' and 3F'-3F''. Again, without chromosomal and membrane markers the interpretation of these images is complicated.

(2) In Fig. 1D and text from pages 5 and 6, the authors compare the proliferative profiles of hearts at 5 dpi, 5 dpi +1 day of culture and, 5 dpi and + 3 days of culture. They observe a decline in the proportion of cardiomyocytes that are cycling in culture and conclude that the media lacks stimuli to maintain cardiomyocyte proliferation or that the media contain inhibitory components. They miss an essential control, which compares the 5 dpi+3 in culture with hearts at 8 dpi (no culture).

Additionally, there is no reference in the text to the uninjured and uninjured +
culture condition, as shown in the graph from 1D.

(3) Protocol - Because this publication is likely to serve as the foundation for the work of others, a detailed protocol is essential. The authors provide a somewhat detailed protocol, but I think this could be improved. In what volume are they doing their culture? What concentration of Glutamax and heparin are they using? Are they floating the sections in the vibratome in water of any other solution? Are they using CO2 in the incubator for these sections? How long are they letting the agarose blocks jellify? What vibratome are they using?

(4) Page 6 - When discussing the advantages of using ex vivo slices, the authors mention that this technique would be useful to study pharmacological perturbations that would otherwise have detrimental effect on the health of the fish. Please, provide specific examples of such pharmacological perturbations.

Minor points:

Page 3 - "Adult hearts show rapid necrosis of the inner myocardium (Kikuchi et al., 2011)". Kikuchi and colleages did not address whether the inner myocardium died by apoptosis or necrosis. They

© 2021. Published by The Company of Biologists under the terms of the Creative Commons Attribution License (https://creativecommons.org/licenses/by/4.0/).

referred to “internal infarcts” and showed that these areas were DAPI-negative. I would advise to avoid using “necrosis” in this context.

Page 5 - In connexion with my previous point from page 3. The authors claim that they did not observe “necrosis”, but in reality, they just check whether there are alterations in the DAPI distribution. To test for necrosis, they would need to use a different technique (i.e., exposing the samples, prior to fixation, to anti-MF20 antibody, which would recognize the exposed sarcomeres from necrotic cells).

Page 5 - I do not observe the pattern that the authors describe in the nppa:Citrine line (low levels of Citrine in ventricular cardiomyocytes and elevated in border zone myocardium). When looking at Fig. 1B and 1C, the nppa:Citrine expression is quite strong in all the trabecular myocardium, not only in the border zone myocardium. This is obvious just by looking at the embryonic myosin staining — the N2.261 is well defined in the border zone, while the citrine staining is equally distributed in all trabecular cells.

Page 12 - The anti-N2.261 from DSHB is a monoclonal, not a polyclonal.

First revision

Author response to reviewers' comments

Reviewer 1
Advance Summary and Potential Significance to Field:
Honkoop et al developed an ex vivo culture system for zebrafish cardiac slices that allows for timelapse imaging of and delicate chemical perturbation of regenerating myocardium. This system has the potential to improve ex vivo screens of zebrafish cardiomyocytes in response to various injury, mechanical, and chemical conditions; while avoiding the necrotic complications of some earlier ex vivo systems.

Reviewer 1 Comments for the Author:
We here raise a few minor comments that may clarify the paper for readers before acceptance.

First of all, we thank the reviewer for his/her time and efforts to review our manuscript. We took all comments of this reviewer to heart and made changes to the manuscript accordingly. Our responses to the reviewer’s comments are given in a point-by-point fashion below, repeating each of the reviewer’s comments, directly followed by our response in coloured text. Changes made to the manuscript appear in a distinct colour in the revised manuscript.

Minor Comments:

1) The authors note that the cardiac slices can be cultured ex vivo for at most three days. They hypothesize that the protocol could be modified to extend the longevity of the culture but do not conduct any further experiments to assess these hypotheses. The authors may want to consider culturing the slices with an air liquid interface (growing them in a trans well) to extend the culture longevity.

Response: We appreciate the suggestion raised by the reviewer. We have cultured the cardiac slices for longer than 3 days (up to 5 days) without any clear signs of deterioration of the tissue. We believe that survival of cardiac slices is not the main limitation of cardiac slice cultures as presented in this manuscript, but to maintain cellular processes that are relevant to study such as cell proliferation. Hence, we believe that future studies should focus on extending the proliferative window in cardiac slice cultures by adjusting culture conditions. We have added this to the manuscript on page 6:
‘*Future studies should focus on optimizing culture conditions by modifying the media composition*

© 2021. Published by The Company of Biologists under the terms of the Creative Commons Attribution License (https://creativecommons.org/licenses/by/4.0/).

or the matrix in which the slices are embedded to prolong the time that cardiomyocyte proliferation is maintained. '

2) In Fig 1B, using the color cyan overlapping with the yellow mCitrine produces a white color that does not stand out dramatically from yellow. Instead of cyan, can another color be used that "pops out" more from the yellow mCitrine background in the merged panels?

As suggested by the reviewer we have changed the colors in Fig.1B to green (nppa:mCitrine) and magenta (N2.261 antibody). Overlap of the two colors now results in a clear, white color.

3) In Fig 2B, arrows indicating border zone vs remote zone could be helpful to the reader -

To clarify the overview picture in Fig.2B, we have now added a yellow dotted line, indicating the injury area. Both zoom-ins are taken from the border zone region (defined as a 200um region from the injury area).

also, in 2F' is there a way to enhance the magenta signal to increase its contrast when overlapped with green?

In this revised version we have replaced old Fig,2F', which showed images of Tg(*myl7:dsRed;nppa:mCitrine*), with new images on Tg(*myl7:dsRed;myl7:BFP-CAAX*) cardiac slices. The Tg(*myl7:BFP-CAAX*) marks the cell membranes and made it easier to see the outline of the cardiomyocytes and to look whether cytokinesis occurred. We used cyan (membrane) and red (nuclei) to get a better contrast in the overlays.

4) Units should be indicated in the vibratome velocity in "Cardiac slice culture" methods section.

We have added units to the vibratome velocity in the "Cardiac slice culture"(p11) methods section. In addition, we have further expanded this section as well as the section "Time- lapse imaging"(p12) to facilitate reproduction of the methods by others.

5) MG-132 also initiates apoptosis. Please discuss how this may contribute to the decrease in cardiomyocyte divisions observed.

Indeed several studies showed that MG-132 can induce apoptosis (PMIDs:14584782, 22897979 and 18795123), however all these studies were performed on tumor cells. We have added this to the discussion on page 9:' *Future studies are required to investigate whether impaired cell cycle progression is the result of persisting sarcomeres or of other factors such as a block in cyclin B proteolysis or induction of apoptosis. '*

6) Please specify what "frequency" is in Figures 2 and 3.

We have changed the labels of the y-axes in fig.2 and 3 from "Frequency" to "# Divisions". This number indicates the cumulative number of proliferative events we observed in our time-lapse movies (n=17). More information on how we quantified these events can be found in methods section "Quantification of proliferative events" (p12-13).

Reviewer 2

Advance Summary and Potential Significance to Field:

In this manuscript, Honkoop et al. developed a cardiac slice culture system to monitor myocardium regeneration after heart injury. The system maintains CM proliferation for 2 days and is adaptable for live imaging and chemical manipulations. The authors further showed an application of the system by showing that proteasome and calpain inhibition blocks sarcomere disassembly and proliferation. The new system is potentially of high interest to the field. However, the study is largely preliminary and has issues with data quality. It does not provide novel information.

We thank the reviewer for his/her time and efforts to review our manuscript. We took all

© 2021. Published by The Company of Biologists under the terms of the Creative Commons Attribution License (https://creativecommons.org/licenses/by/4.0/).

comments of this reviewer to heart and made changes to the manuscript accordingly. Our responses to the reviewer's comments are given in a point-by-point fashion below, repeating each of the reviewer's comments, directly followed by our response in coloured text. Changes made to the manuscript appear in a distinct colour in the revised manuscript.

Reviewer 2 Comments for the Author:
Major:
1. The authors failed to demonstrate applications of the system. It is of no surprise that MG132 induces cell cycle arrest. Known signaling pathways that regulate heart regeneration should be tested instead, for instance, by inhibiting the Fgf pathway or treating with Alfacalcidol.

Response:
The main advance of the cardiac slice system is that it allows for live imaging of cellular processes deep inside the zebrafish heart, which are otherwise inaccessible. We demonstrated the application of this method by live-imaging of injury-induced cardiomyocyte nuclear divisions, cytokinesis and sarcomere disassembly and reassembly. From these observations we conclude that cardiomyocytes in the adult heart have at least two different mechanisms for organizing sarcomere structures during cell division. First, cardiomyocytes can have completely disassembled sarcomeres before, during and after cell division. Second, cardiomyocytes can transiently and partially disassemble and reassemble sarcomeres during cell division. For the latter process, the proteasome inhibitor (MG-132) point to a model in which ubiquitin mediated sarcomere breakdown around the nucleus allows prometaphase cells to progress through mitosis.
As such, the cardiac slice cultures in combination with live imaging form a valuable tool to study the dynamics of cellular processes such as sarcomere disassembly and reassembly in adult cardiomyocytes.

The reviewer asked if cardiomyocyte proliferation in the cardiac slices is affected by adding Alfacalcidol (10 μM) to the culture medium. To address this, we incubated either injured cardiac slices for 24 hours or uninjured cardiac slices for 48 hours with Alfacalcidol (10 μM) and analysed cardiomycyte proliferation, but we observed no effect (Fig.R1.1).

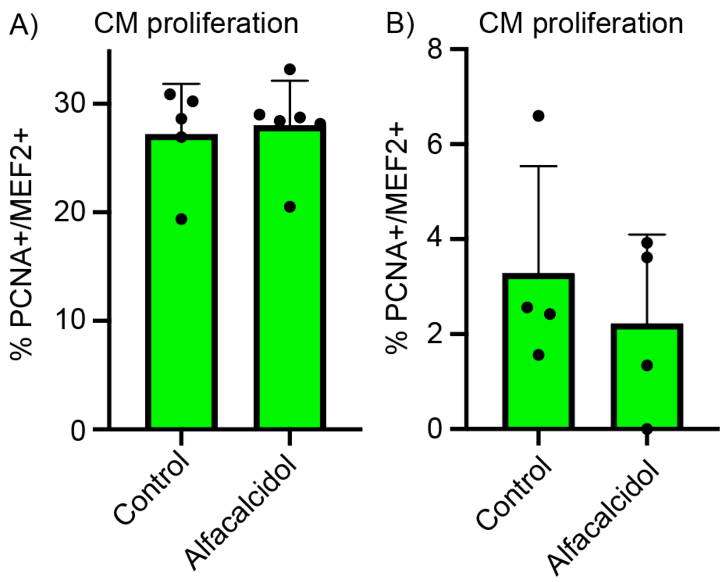

Figure R1.1: The effect of alfacalcidol (10 μM) on cardiomyocyte proliferation. (A) Quantification of the percentage of proliferating cardiomyocytes in cardiac slice cultures of 5dpi hearts after 24 hours of culture. (B) Quantification of the percentage of proliferating cardiomyocytes in cardiac slice cultures of uninjured hearts after 48hours of culture.

2. BDM is a myosin II inhibitor that may arrest the cell cycle and block cytokinesis. BDM inhibits contraction of the contractile ring of the cleavage furrow during cytokinesis. It is unclear how the system mimics the in vivo situation and whether the claimed cytokinesis is reliable.

© 2021. Published by The Company of Biologists under the terms of the Creative Commons Attribution License (https://creativecommons.org/licenses/by/4.0/).

We use BDM in the culture medium to inhibit cardiomyocyte contractions that would otherwise interfere with the live imaging. Although it is generally believed that BDM inhibits all myosins, there are several reports demonstrating that some myosins, including non- muscle myosins, are not inhibited by BDM (PMIDs: 12630704, 11744924, 12563270). In addition cytokinesis is not solely depending on myosin activity. Although cytokinesis typically relies on actomyosin interactions and the contractile stress that this generates, it has been described that other redundant mechanisms can generate sufficient forces and take over the role of myosins in culture (PMIDs: 15944220, 27505246).
As the reviewer pointed out that cytokinesis was difficult to assess in our images with the cytoplasmic GFP signal of the Tg(*nppa:mCitrine*)(see also point#4), we now imaged cardiomyocyte proliferation using a transgene that marks the cell membrane Tg(*myl7:BFP- CAAX*). We performed live imaging on cardiac slices from Tg(*myl7:BFP-CAAX;myl7:dsRed*) fish to simultaneously label the cardiomyocyte cell membrane and nucleus (Data presented in new Fig.2F and F'). Importantly, we find that in cardiomyocytes nuclear division is rapidly followed by the permanent separation of the two new daughter nuclei by the plasma membrane, indicating that cytokinesis occurs in the cultured cardiac slices.

3. CM proliferation was primarily observed in the first 2 days of culture. These division events are likely ongoing proliferation at the time of heart isolation. It is possible that no new CM proliferation was initiated during culture. If true, it reduces the value of the system.

The reviewer rightly notices the sharp decrease in proliferation we observe in cardiac slices during culture. In order to gain more in-depth knowledge on the proliferation that occurs during culture, we now performed EdU incorporation experiments during culture (Fig.S2). Therefore we added EdU to the cardiac slices at the start of the culture (t=0h), at 6 hours after culture or at 24 hours after culture. After imaging and quantification we observed a significant amount of cells that had incorporated the EdU when added at t=0h and t=6h and only very few cells that had incorporated the EdU when this was added at t=24h to the culture. From this we conclude that cells in cardiac slices actively synthesize new DNA in the S-phase of the cell cycle during the first day in culture.

These new results are consistent with the results from the PCNA labeling presented in the original version of the manuscript (Fig.1D) and indeed suggest that the observed cardiomyocyte proliferation is induced before heart isolation. Although this somewhat limits the use of cardiac slice cultures, it is a valuable tool to study cellular dynamics during cardiomyocyte proliferation within its native tissue context such as sarcomere disassembly and reassembly. We have included this limitation to the discussion on page 6:
'*EdU incorporation during cardiac slice culture confirmed cells were actively cycling during the first 24 hours of culture after which proliferation in the slices ceased (Fig.S2). Future studies should focus on optimizing culture conditions by modifying the media composition or the matrix in which the slices are embedded to prolong the time that cardiomyocyte proliferation is maintained.* '

4. Most videos and images are blurry. A time interval of 30-45 min is way too long. A shorter window of 5-10 min is necessary to visualize cellular details of division.

We agree with the reviewer that time-lapse imaging would benefit from smaller time intervals, as to capture cellular events during proliferation in more detail. In the revised vision of the manuscript we have added time-lapse movies of proliferating cardiomyocytes with a time interval of 10 (Fig.3 and Movies 5 and 6) and 15 minutes (Fig.2F, F', Fig.S4 and Movies 4 and 7) respectively. This allowed us to gain further knowledge on sarcomere disassembly and the duration of the M-phase of a cell.

As the cardiac slices are 200 micrometer thick and we wanted to capture processes occurring inside the cardiac slice, the live imaging was performed on confocal microscope using a 20x objective with a large working distance. Due to the low magnification lens, digital zoom in was required to see the structures which caused some blurring of the images. As some blurring is also caused by the PDF conversion we would like to refer to the high-res images of the figures that we uploaded together with the manuscript.

© 2021. Published by The Company of Biologists under the terms of the Creative Commons Attribution License (https://creativecommons.org/licenses/by/4.0/).

The dynamics of sarcomere structure is potentially a novel discovery; however, the claims are not supported by the blurry images and arbitrary outlines and quantifications.

In the original manuscript the images in Fig.3 (showing sarcomere dynamics) were made using the Tg(*myl7:*actn3b-EGFP). We have now replaced all images in Fig.3 with new images using Tg(Tg(*myl7:*actn3b-EGFP; *myl7*:DsRed). This allowed us to simultaneously image nuclear division and sarcomere dynamics. In addition, we were now able to use the cytoplasmic fraction of the *myl7*:dsRed signal after nuclear envelope breakdown as a proxy of the cellular outline, which we have used to indicate cell borders during nuclear division. Due to the almost complete disassembly of sarcomeres in cardiomyocyte adjacent to the injury area, the mainly cytoplasmic actn3b-GFP signal appears blurry. In the cardiomyocytes distal from the injury (Fig.3E, E') the actn3b-GFP signal shows clear striations indicating the z-discs of the sarcomeres. For best representation of the data we would like to refer to the high-resolution images that we uploaded together with the manuscript.

To confirm that cytokinesis occurs, we now included results from live imaging on a new Tg(*myl7:BFP-CAAX;myl7:actn3b-GFP*) line (Fig.S4 and Movie 7), which simultaneously marks both the membranes and sarcomeres in cardiomyocytes. These results confirm cardiomyocyte cytokinesis in the cardiac slices.

In Figure 4H and Movie 10, the temporal resolution does not allow me to track these fragments.

We have removed the tracking of sarcomeres in this context from the revised manuscript. Instead, we have added more stills of Fig.4G and Movie 8 and replaced the star in Fig.4G which covered the fragment itself by an arrow on top of it.

5. The authors' claim that the "use of PCNA as a marker for cardiomyocyte proliferation may result in an overestimation of the actual number of cell divisions" is questionable. PCNA expression increases from the late G1 phase through the S-phase of the cell cycle. Theoretically, PCNA staining marks proliferating cells from the late G1 to M phase and labels more cells than the G2/M phase marker pH3 and the M phase sign of nuclear division. Unless every single proliferating cell was followed from the G1 phase to the M phase, there is no evidence to support this claim in the current study. On the other hand, the authors' observation may suggest that cell cycle progression may be blocked in some CMs (PCNA+ but never divide). The FUCCI reporters or long-term EdU incorporation can be used to clarify this.

As suggested by the reviewer we have now performed EdU incorporation assays on the cardiac slices, which results are presented in Supplementary Figure 2 and results are described on page 6. Briefly, these results indicate that cardiomyocytes are able to actively initiate the S-phase during the first 24 hours of culture. Additionally, we performed a co- staining for PCNA and EdU to shed more light on the overrepresentation of proliferative cells by PCNA (Fig.R2.1). This shows that approximately 70% of all PCNA+ cells after 24 hours of culture had incorporated EdU.

© 2021. Published by The Company of Biologists under the terms of the Creative Commons Attribution License (https://creativecommons.org/licenses/by/4.0/).

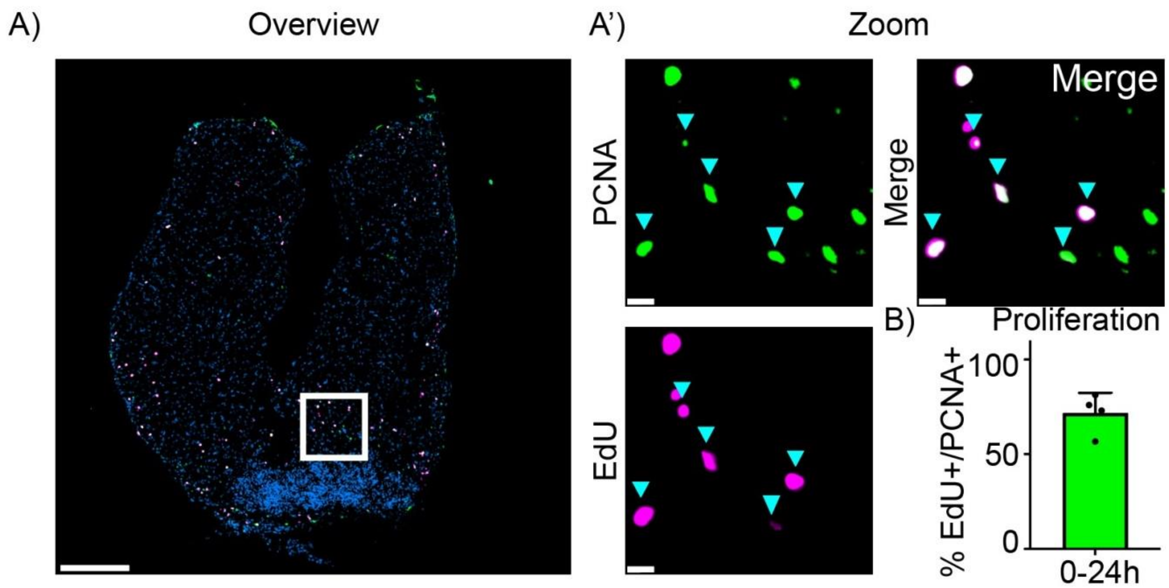

Figure R2.1: EdU uptake by PCNA positive cells. (A, A') Overview (A) and Zoom-ins (A') of an immunolabeling for DAPI (blue, overview only), PCNA (green) and EdU (magenta). Scale bars indicate 200 µm (overview) or 10 µm (zoom) (B) Quantification of the percentage of PCNA+ cells also positive for EdU+.

Based on these new results we have removed the initial claim that the use of PCNA as a marker for cardiomyocyte proliferation may result in an overestimation of the actual number of cell divisions.

6. This study focused on CM proliferation. It would be informative to show whether other cells (i.e., epicardial cells, endocardial cells, endothelial cells) proliferate?

To address this we performed a 24 hour EdU incorporation assay on cultured cardiac slices and co-stained these cardiac slices for EdU, MEF2 (marking cardiomyocyte nuclei) and DAPI (marking all nuclei) (Fig.S2). We observed that about half of the cells that had incorporated EdU were negative for MEF2 demonstrating that cell proliferation in cardiac slices was not restricted to cardiomyocytes. We were not able to identify which cells types are proliferating and which cell types may not proliferate as we lacked appropriate markers for all different cell types that are present in the heart.

We added this to the manuscript on page 6:
'*Cell proliferation was also observed in non-cardiomyocytes (Fig.S2).*'

Figure S1A. Why were the outflow tract and atrium kept in the 5-dpi culture but not in others? Were these regions counted in B? Were the blood clot regions measured? There are many blood cells in these regions that may affect the conclusion if they are counted.

The Fig.S1 have been replaced by new images that also include TUNEL staining. The 5dpi control hearts were not cultured but directly fixed after heart extraction, Therefore, the outflow tract and atrium were not removed from these hearts. All quantifications on these stainings were performed in the trabecular myocardium of the ventricle.

The authors should separate cell types (e.g., CMs, endocardial cells, epicardial cells).

As indicated above we were not able to identify different cells types in the cardiac slices as we lacked appropriate markers for all different cell types that are present in the heart.

The authors claimed that no necrosis was observed. However, there is no clear data to prove this.

© 2021. Published by The Company of Biologists under the terms of the Creative Commons Attribution License (https://creativecommons.org/licenses/by/4.0/).

Also, is there apoptosis?

It is true that the claims made in the original manuscript were insufficiently supported by the data presented. In order to address whether cell death occurs we have now included TUNEL stainings on cardiac slices and compared this to whole heart cultures and uncultured control hearts. These data are presented in the new Fig.S1. As we setup the cardiac slices to be able to study trabecular cardiomyocytes in more detail, we quantified cell death in the inner myocardium. Importantly, we observed that cell death in cardiac slices was much lower compared to whole heart cultures. Although low percentages of cell death were detected in the cardiac slice cultures, this did not result in a significant drop of cardiomyocyte density. Thereby, cardiac slices are an excellent tool to perform live imaging on cardiomyocytes within their native tissue context.

7. Figure 2F'. The schematic of cell shapes is very arbitrary. The cell boundaries are not evident in the video. A membrane maker seems necessary.

As suggested by the reviewer we have now performed live imaging experiments on a *Tg*(*myl7:NucDsRed;myl7:BFP-CAAX*) line, which marks both the nuclei and the membrane of cardiomyocytes. All images in Fig.2F' have been replaced.

8. Figure 3EF. The distal regions in both figure panels contain both well-organized and less-assembled sarcomeres. I cannot draw a clear conclusion from these two images. Again, the outlining of cell boundaries in panel F' is very arbitrary.

This has been addressed in point#4 above:
In the original manuscript the images in Fig.3 (showing sarcomere dynamics) were made using the *Tg(myl7:actn3b-EGFP)*. We have now replaced all images in Fig.3 with new images using *Tg(myl7:actn3b-EGFP; myl7:DsRed)*. This allowed us to simultaneously image nuclear division and sarcomere dynamics. In addition, we were now able to use the cytoplasmic fraction of the *myl7*:dsRed signal after nuclear envelope breakdown as a proxy of the cellular outline, which we have used to indicate cell borders during nuclear division.
Due to the almost complete disassembly of sarcomeres in cardiomyocyte adjacent to the injury area, the mainly cytoplasmic actn3b-GFP signal appears blurry. In the cardiomyocytes distal from the injury (Fig.3E,E') the actn3b-GFP signal shows clear striations indicating the z- discs of the sarcomeres. For best representation of the data we would like to refer to the high-resolution images that we uploaded together with the manuscript.

9. Figure 3E'. The images do not bear enough resolution to show completed cytokinesis.

To confirm that cytokinesis occurs during the sarcomere behavior described in Fig.3, we now included results from live imaging on a new Tg(*myl7:BFP-CAAX;myl7:actn3b-GFP*) line (see Fig.S4), which simultaneously marks both the membranes and sarcomeres in cardiomyocytes. These results confirm cardiomyocyte cytokinesis in the cardiac slices.

10. Figure 4BC. Why were those regions chosen for the quantifications?

Cardiomyocyte proliferation was accompanied by highly dynamic sarcomeres surrounding the dividing nucleus (Fig.3E, E'). Hence, we hypothesized that quick and localized degradation of sarcomeres would be pivotal for proper cardiomyocyte proliferation. This is also clarified in the text on page 9. Previously it has been shown that prior to nuclear division a ubiquitin cloud arises around the nucleus, which might target structures in the proximity for degradation (PMID: 15226401). As we hypothesized that the proteasome and calpains might be important for this localized breakdown, we chose to inhibit these using MG-132 and quantify this local disassembly of sarcomeres surrounding the nucleus.

Minor:
11. Figure 1D. Quantification of MEF2+/PCNA+ cells was done in the border zone of injured hearts/cardiac slices but in the remote area of uninjured hearts/cardiac slices. Why were different regions counted?

© 2021. Published by The Company of Biologists under the terms of the Creative Commons Attribution License (https://creativecommons.org/licenses/by/4.0/).

As the border zone holds most of the regenerative capacity in the injured heart, we were most interested in cardiomyocyte proliferation in the border zone.
In order to exclude the possibility that the observed proliferation is induced by the vibratome sectioning or the culturing conditions we also quantified proliferation in a cardiac slice that is cultured in the same manner but lacks the context of an injury. Interestingly we observed that in this context, no proliferation was observed. Indicating proliferation in the border zone of cardiac slices is dependent on the injury context and not an artefact of culturing the cardiac slices.

Reviewer 3
Advance Summary and Potential Significance to Field:
In this manuscript, Honkoop and colleagues describe an adult zebrafish cardiac slice system to study cardiomyocyte proliferation ex vivo. The authors collect adult hearts after cryoinjury, include them in agarose and prepare slices using a vibratome. These cardiac slices can be cultured for a few days with better tissue preservation than other methods that use the whole ventricle. The authors propose this system as a new opportunity to study cell division ex vivo, as they provide evidence that CMs continue to divide in the slices.
Importantly, this technique allows them to analyze the image cytokinesis and sarcomere disassembly ex vivo.

As noted below, I find that most of the conclusions related to cytokinesis, sarcomeric disassembly etc., might be premature and require further analysis using more specific transgenic lines. However, I find that the present manuscript may be of interest to the regeneration community from a methodological perspective. I find that the text is well written, easy to follow, and appropriately referenced.

First of all, we thank the reviewer for his/her time and efforts to review our manuscript. We took all comments of this reviewer to heart and made changes to the manuscript accordingly. Our responses to the reviewer's comments are given in a point-by-point fashion below, repeating each of the reviewer's comments, directly followed by our response in coloured text. Changes made to the manuscript appear in a distinct colour in the revised manuscript.

Reviewer 3 Comments for the Author:
Major issues:
(1) Some of the conclusions from the sarcomeric dynamic, nuclear division, etc., might be a bit premature. I'm missing some additional tools to support most of these conclusions. For example, although valuable and didactic, the authors have used dashed lines to indicate the border between cells. Inspecting the images -and this might be a limitation of the PDF file provided to the reviewers-, I do not see clear boundaries in most cases. This could be solved using any of the multiple available transgenic lines that express membrane-bound fluorescent proteins in cardiomyocytes. The same is true for some nuclear labelings (see specific examples below). However, I'm not in favor of asking the authors to repeat their experiments with several new transgenes to satisfy this reviewer. Instead, I would strongly recommend adding a paragraph in the text where the authors acknowledge these limitations and discuss future experiments to tackle these gaps.

First of all, we thank the reviewer for his/her time and efforts to review our manuscript. We took all comments of this reviewer to heart and made changes to the manuscript accordingly. Our responses to the reviewer's comments are given in a point-by-point fashion below, repeating each of the reviewer's comments, directly followed by our response in coloured text. Changes made to the manuscript appear in a distinct colour in the revised manuscript.

Specific examples:
Fig. 2F' — The myl7:DsRed line is helpful to identify cardiomyocyte nuclei, but it is most useful in uninjured hearts. In proliferating cardiomyocytes, the DsRed signaling becomes very diffuse (Lepilina et al., 2006; Wills et al., 2008), and the identification of the nuclei becomes very difficult. This "diffuse" pattern is because the DsRed here has an nls sequence, which directs the protein to the nucleus, and it is not a histone-fused protein. An H2B-XFP would be much more appropriate for this task: otherwise, we're missing mitosis altogether. The use of nppa:Citrine is not particularly useful in this context.

© 2021. Published by The Company of Biologists under the terms of the Creative Commons Attribution License (https://creativecommons.org/licenses/by/4.0/).

As the reviewer pointed out that cytokinesis was difficult to assess in our images with the cytoplasmic GFP signal of the Tg(*nppa:mCitrine*)(see also point#4), we now imaged cardiomyocyte proliferation using a transgene that marks the cell membrane Tg(*myl7:BFP-CAAX*). We performed live imaging on cardiac slices from Tg(*myl7:BFP-CAAX;myl7:dsRed*) fish to simultaneously label the cardiomyocyte cell membrane and nucleus (Data presented in new Fig.2F and F'). Importantly, we find that in cardiomyocytes nuclear division is rapidly followed by the permanent separation of the two new daughter nuclei by the plasma membrane, indicating that cytokinesis occurs in the cultured cardiac slices.
We also would like to refer to the high resolution images of the figures that we uploaded with the manuscript.

Fig. 3E' and 3E'' and 3F'-3F''. Again, without chromosomal and membrane markers, the interpretation of these images is complicated.

In the original manuscript the images in Fig.3 (showing sarcomere dynamics) were made using the *Tg(myl7:actn3b-EGFP)*. We have now replaced all images in Fig.3 with new images using *Tg(Tg(myl7:actn3b-EGFP; myl7:DsRed)*. This allowed us to simultaneously image nuclear division and sarcomere dynamics. In addition, we were now able to use the cytoplasmic fraction of the *myl7*:dsRed signal after nuclear envelope breakdown as a proxy of the cellular outline, which we have used to indicate cell borders during nuclear division. Due to the almost complete disassembly of sarcomeres in cardiomyocyte adjacent to the injury area, the mainly cytoplasmic actn3b-GFP signal is very weak. In the cardiomyocytes distal from the injury (Fig.3E,E') the actn3b-GFP signal shows clear striations indicating the z- discs of the sarcomeres.

To confirm that cytokinesis occurs, we now included results from live imaging on a new Tg(*myl7:BFP-CAAX;myl7:actn3b-GFP*) line (see Fig.S4), which simultaneously marks both the membranes and sarcomeres in cardiomyocytes (Fig.S4). These results confirm cardiomyocyte cytokinesis in the cardiac slices.

For best representation of the data we would like to refer to the high-resolution images that are uploaded together with the manuscript.

(2) In Fig. 1D and text from pages 5 and 6, the authors compare the proliferative profiles of hearts at 5 dpi, 5 dpi +1 day of culture and, 5 dpi and + 3 days of culture. They observe a decline in the proportion of cardiomyocytes that are cycling in culture and conclude that the media lacks stimuli to maintain cardiomyocyte proliferation or that the media contain inhibitory components. They miss an essential control, which compares the 5 dpi+3 in culture with hearts at 8 dpi (no culture).

The reviewer rightly points at the importance of 6dpi and 8dpi *in vivo* controls to compare the percentages of 5+1 and 5+3 in cardiac slice cultures to. We have added these data to the revised manuscript. As expected, we observed that proliferation persists in the 8dpi *in vivo* control and that there is a significant difference between these hearts and 5dpi + 3d culture cardiac slices.

Additionally, there is no reference in the text to the uninjured and uninjured + culture condition, as shown in the graph from 1D.

It is true we did not refer to this result. We have added a reference to the results in the revised manuscript.

(3) Protocol - Because this publication is likely to serve as the foundation for the work of others, a detailed protocol is essential. The authors provide a somewhat detailed protocol, but I think this could be improved. In what volume are they doing their culture? What concentration of Glutamax and heparin are they using? Are they floating the sections in the vibratome in water of any other solution? Are they using CO2 in the incubator for these sections? How long are they letting the agarose blocks jellify? What vibratome are they using?

We agree with the reviewer that our work would serve as a foundation for others to build on and study cardiomyocyte proliferation in more detail. We have expanded the protocol in the methods

© 2021. Published by The Company of Biologists under the terms of the Creative Commons Attribution License (https://creativecommons.org/licenses/by/4.0/).

sections "Cardiac slice culture" (p11) and "Time-lapse imaging" (p11-12) and have added more details as to make the protocol more reproducible.

(4) Page 6 - When discussing the advantages of using ex vivo slices, the authors mention that this technique would be useful to study pharmacological perturbations that would otherwise have detrimental effect on the health of the fish. Please, provide specific examples of such pharmacological perturbations.

Many pharmalogical perturbations directed to interfere with general processes have toxic side effects *in vivo*, which might not only harm the fish but also indirectly interfere with the outcome of the study. Examples general processes that can be perturbed by pharmacological interventions include energy metabolism (PMID: 26689325, 11281565), translation (PMID: 23059828, 32373584) and transcription (PMID: 9989808).
We have included these processes in the text on page 6: '*Ex vivo* slice cultures open up the opportunity to study cellular processes in regenerating zebrafish hearts by pharmacological perturbations that would otherwise have detrimental effect on the health of the fish because they interfere with general processes that are important in other cells and organs (e.g. energy metabolism or transcription/translation).'

Minor points:
Page 3 - "Adult hearts show rapid necrosis of the inner myocardium (Kikuchi et al., 2011)". Kikuchi and colleages did not address whether the inner myocardium died by apoptosis or necrosis. They referred to "internal infarcts" and showed that these areas were DAPI- negative. I would advise to avoid using "necrosis" in this context.

We have corrected this and replaced any statements on necrosis in the adult heart now referring to the observed internal infarctions.

Page 5 - In connexion with my previous point from page 3. The authors claim that they did not observe "necrosis", but in reality, they just check whether there are alterations in the DAPI distribution. To test for necrosis, they would need to use a different technique (i.e., exposing the samples, prior to fixation, to anti-MF20 antibody, which would recognize the exposed sarcomeres from necrotic cells).

In the revised version of the manuscript we have added TUNEL stainings on cardiac slices cultures and whole heart cultures (Fig.S1). TUNEL stains fragmented DNA caused either by apoptosis or necrosis (in the text referred to as "cell death"). As expected, whole heart cultures showed strong TUNEL staining and loss of MEF2 signal inside the heart. In the cardiac slice cultures we observed that the percentage of TUNEL positive cell was strongly reduced compared to whole heart cultures. Although low percentages of cell death were present in cardiac slice cultures, this did not result in a significant decrease in MEF2+ density in the inner myocardium. Thereby, cardiac slices are an excellent tool to perform live imaging on cardiomyocytes within their native tissue context.

Page 5 - I do not observe the pattern that the authors describe in the nppa:Citrine line (low levels of Citrine in ventricular cardiomyocytes and elevated in border zone myocardium).
When looking at Fig. 1B and 1C, the nppa:Citrine expression is quite strong in all the trabecular myocardium, not only in the border zone myocardium. This is obvious just by looking at the embryonic myosin staining — the N2.261 is well defined in the border zone, while the citrine staining is equally distributed in all trabecular cells.

It is correct that nppa:mCitrine is also expressed in the remote trabeculated myocardium. However, border zone cardiomyocytes typically show elevated levels of this transgene (PMID:31868166). As this was not well visible in the original figure we replaced the images and changed the colors to green (mCitrine) and magenta (N2.261).

Page 12 - The anti-N2.261 from DSHB is a monoclonal, not a polyclonal.

© 2021. Published by The Company of Biologists under the terms of the Creative Commons Attribution License (https://creativecommons.org/licenses/by/4.0/).

We would like to thank the reviewer for pointing this out and we have replaced the word polyclonal by the word monoclonal in the revised manuscript (p12).

Second decision letter

MS ID#: DEVELOP/2021/199740

MS TITLE: Live imaging of adult zebrafish cardiomyocyte proliferation ex vivo

AUTHORS: Hessel Honkoop, Phong D Nguyen, Veronique E.M. van der Velden, Katharina F Sonnen, and Jeroen Bakkers
ARTICLE TYPE: Research Report

I am happy to tell you that your manuscript has been accepted for publication in Development, pending our standard ethics checks.

Reviewer 1

Advance summary and potential significance to field

The authors have adequately addressed my concerns, therefore I would recommend acceptance for publication. Congratulations to the authors on developing this imaging platform which could benefit many researchers in the field of heart regeneration.

Comments for the author

None.

Reviewer 2

Advance summary and potential significance to field

The authors have addressed my concerns. I do not have further comments.

Comments for the author

The authors have addressed my concerns. I do not have further comments.

Reviewer 3

Advance summary and potential significance to field

The authors report here a new system to study cardiac slices ex vivo. I anticipate several labs using this technique to perform ex vivo imaging during and understand sarcomere disassembly, cytokinesis, etc.

Comments for the author

The authors have addressed all my comments. They have added a number of figures that strengthen their conclusions. I'm most enthusiastic about this new method and congratulate the authors on a very good job.

© 2021. Published by The Company of Biologists under the terms of the Creative Commons Attribution License (https://creativecommons.org/licenses/by/4.0/).
